# Global disability-adjusted life-year estimates of long-term health burden and undernutrition attributable to diarrhoeal diseases in children younger than 5 years

**DOI:** 10.1016/S2214-109X(18)30045-7

**Published:** 2018-02-09

**Authors:** Christopher Troeger, Danny V Colombara, Puja C Rao, Ibrahim A Khalil, Alexandria Brown, Thomas G Brewer, Richard L Guerrant, Eric R Houpt, Karen L Kotloff, Kavita Misra, William A Petri, James Platts-Mills, Mark S Riddle, Scott J Swartz, Mohammad H Forouzanfar, Robert C Reiner, Simon I Hay, Ali H Mokdad

**Affiliations:** a Institute for Health Metrics and Evaluation, University of Washington, Seattle, WA, USA; b Big Data Institute, Li Ka Shing Centre for Health Information and Discovery, University of Oxford, Oxford, UK; c Global Enterics, LLC, Seattle, WA, USA; d Center for Global Health, Division of Infectious Diseases and International Health, University of Virginia, Charlottesville, VA, USA; e Division of Infectious Diseases and International Health, University of Virginia, Charlottesville, VA, USA; f Departments of Pediatrics and Medicine, Center for Vaccine Development, University of Maryland School of Medicine, Baltimore, MD, USA; g Bureau of HIV/AIDS Prevention and Control, New York City Department of Health and Mental Hygiene, New York, NY, USA; h Uniformed Services University of the Health Sciences, Bethesda, MD, USA

## Abstract

**Background:**

Diarrhoea is a leading cause of death and illness globally among children younger than 5 years. Mortality and short-term morbidity cause substantial burden of disease but probably underestimate the true effect of diarrhoea on population health. This underestimation is because diarrhoeal diseases can negatively affect early childhood growth, probably through enteric dysfunction and impaired uptake of macronutrients and micronutrients. We attempt to quantify the long-term sequelae associated with childhood growth impairment due to diarrhoea.

**Methods:**

We used the Global Burden of Diseases, Injuries, and Risk Factors Study framework and leveraged existing estimates of diarrhoea incidence, childhood undernutrition, and infectious disease burden to estimate the effect of diarrhoeal diseases on physical growth, including weight and height, and subsequent disease among children younger than 5 years. The burden of diarrhoea was measured in disability-adjusted life-years (DALYs), a composite metric of mortality and morbidity. We hypothesised that diarrhoea is negatively associated with three common markers of growth: weight-for-age, weight-for-height, and height-for-age *Z*-scores. On the basis of these undernutrition exposures, we applied a counterfactual approach to quantify the relative risk of infectious disease (subsequent diarrhoea, lower respiratory infection, and measles) and protein energy malnutrition morbidity and mortality per day of diarrhoea and quantified the burden of diarrhoeal disease due to these outcomes caused by undernutrition.

**Findings:**

Diarrhoea episodes are significantly associated with childhood growth faltering. We found that each day of diarrhoea was associated with height-for-age *Z*-score (–0·0033 [95% CI −0·0024 to −0·0041]; p=4·43 × 10^−14^), weight-for-age *Z*-score (–0·0077 [–0·0058 to −0·0097]; p=3·19 × 10^−15^), and weight-for-height *Z*-score (–0·0096 [–0·0067 to −0·0125]; p=7·78 × 10^−11^). After addition of the DALYs due to the long-term sequelae as a consequence of undernutrition, the burden of diarrhoeal diseases increased by 39·0% (95% uncertainty interval [UI] 33·0–46·6) and was responsible for 55 778 000 DALYs (95% UI 49 125 400–62 396 200) among children younger than 5 years in 2016. Among the 15 652 300 DALYs (95% UI 12 951 300–18 806 100) associated with undernutrition due to diarrhoeal episodes, more than 84·7% are due to increased risk of infectious disease, whereas the remaining 15·3% of long-term DALYs are due to increased prevalence of protein energy malnutrition. The burden of diarrhoea has decreased substantially since 1990, but progress has been greater in long-term (78·7% reduction [95% UI 69·3–85·5]) than in acute (70·4% reduction [95% UI 61·7–76·5]) DALYs.

**Interpretation:**

Diarrhoea represents an even larger burden of disease than was estimated in the Global Burden of Disease Study. In order to adequately address the burden of its long-term sequelae, a renewed emphasis on controlling the risk of diarrhoea incidence may be required. This renewed effort can help further prevent the potential lifelong cost on child health, growth, and overall potential.

**Funding:**

Bill & Melinda Gates Foundation.

## Introduction

Measurement of prevalence, incidence, and mortality associated with diarrhoeal diseases is important for policy decisions, resource allocation, and targeted interventions, but it might not provide a complete understanding of the true disease burden. Evidence is increasing that childhood diarrhoea, especially in the first 2 years of life, can negatively affect nutrient absorption, leading to poor physical growth.^1^, ^2^ Additional measures of long-term health loss due to diarrhoea, other than incidence and mortality, are needed. Disability-adjusted life-years (DALYs) are designed to quantify both acute and long-term health burden and are the sum of years of life lost due to premature mortality and years lived with disability.^3^ Since their introduction more than 20 years ago, DALYs have become a key metric for assessing and monitoring population health and setting priorities within a nation's health sector.^3^

**Research in context**

**Evidence before this study**

We searched PubMed for articles without language or publication date restrictions for (“stunting” OR “wasting” OR “growth” OR “malnutrition”) AND (“diarrhea”) AND (“prospective” OR “case control” OR “trial” OR “cohort”) originally on May 2, 2017, with an update on Jan 10, 2018. We identified 198 publications, including 17 that provided information about the association between diarrhoea and childhood growth. Many of these studies found an association between diarrhOea, measured as prevalence or cumulative exposure, and childhood growth indicators but varied in exposure and outcome definitions. We also identified several publications that reviewed this association, but none attempted to systematically quantify the burden of diarrhoea on childhood growth.

**Added value of this study**

Our study provides a systematic, global set of estimates of the effect of diarrhoea on childhood growth impairment and the burden of impaired growth, measured in disability-adjusted life-years. We assess how diarrhoea affects the risk of subsequent infectious diseases and quantify these outcomes for each country, both sexes, and every 5 years since 1990 for children younger than 5 years. These estimates suggest that the burden of diarrhoea among children under 5 years old is multifaceted, causing increases in acute and long-term disability-adjusted life-years, and was previously under-recognised. After accounting for diarrhoea and a risk factor for childhood growth faltering, the number of DALYs due to diarrhoea among children under-5 increased by about 40%.

**Implications of all the available evidence**

Interventions and policy to prevent diarrhoea incidence could improve child growth and infectious disease burden beyond diarrhoea exclusively. Childhood growth and infectious diseases have a complicated, circular relationship. Reducing the risk of diarrhoea might have broad effects on childhood growth and ability to thrive. Our study findings will guide public health policy for prioritising resource allocation for alleviating the burden of diarrhoeal diseases.

The Global Burden of Diseases, Injuries, and Risk Factors Study (GBD) is a comprehensive and systematic effort to quantify deaths and incidence, prevalence, and DALYs for 264 diseases, including diarrhoea. Despite a substantial reduction of 65% in diarrhoea mortality among children younger than 5 years since 1990, the decline of diarrhoea incidence among this age group has been less pronounced than that of mortality, decreasing by 24% in the same time period and remaining the fourth-leading cause of under-5 DALYs in 2015.^4^, ^5^, ^6^

In this study, we leverage the Global Burden of Diseases, Injuries, and Risk Factors Study 2016 (GBD 2016) to update the burden of childhood diarrhoea, accounting for its long-term health outcomes due to undernutrition. We conceptualise this pathway by first estimating the effect per day of diarrhoea on three common measures of child growth. Undernutrition is well established as an important risk factor for infectious disease morbidity and mortality and is necessary for protein energy malnutrition (PEM). By evaluating the change in undernutrition, we attribute a fraction of all DALYs associated with undernutrition and PEM from GBD 2016 to diarrhoea. It is important to measure chronic health consequences to assess true health burden, which will in turn inform policy and decision making regarding the reduction of diarrhoeal burden through primary or secondary prevention strategies.

## Methods

### Overview

This study has five components. The DALYs due to acute diarrhoea, childhood undernutrition, and PEM were previously estimated as part of GBD 2016. This analysis consists of a systematic literature review and meta-analysis, quantification of DALYs attributable to diarrhoea via growth impairment, and quantification of DALYs attributable to diarrhoea due to PEM. The final step in the estimation of the total burden of diarrhoea, accounting for long-term outcomes associated with growth impairment, is linking these results to the larger GBD framework.

### GBD methods

This study leveraged previously published estimates from GBD 2016. GBD provides comprehensive and internally consistent epidemiological estimates for more than 300 causes of death and disability and 90 risk factors for 195 locations, by year, sex, and four age groups younger than 5 years. Detailed descriptions of all GBD methods have been previously published.^6^, ^7^, ^8^, ^9^, ^10^ This study used two main inputs from GBD 2016. The first was the incidence of diarrhoea, estimated with use of DisMod-MR 2.1 and data from representative population surveys, health-care utilisation records, and the scientific literature. The second was the number of DALYs attributable to three childhood undernutrition indicators: low height-for-age, low weight-for-age, and low weight-for-height. Childhood undernutrition is a risk factor for three outcomes in GBD: diarrhoea, lower respiratory infections, and measles. The DALYs attributable to undernutrition were based on the prevalence of the undernutrition indicators and the association between those indicators and the outcomes. Additionally, PEM is entirely attributable to low weight among children younger than 5 years in GBD 2016. More detail is given in the appendix and the Global Burden of Diseases, Injuries, and Risk Factors Study 2015 diarrhoea,^6^ GBD 2016 causes of death,^7^ GBD 2016 non-fatal,^8^ and GBD 2016 risk factors^9^ publications.

### Effect size and systematic review

This extended analysis began with the hypothesis that diarrhoea is causally associated with childhood growth failure on the basis of biological plausibility and evidence from the scientific literature.^11^, ^12^ We did a series of systematic reviews to establish the change in *Z*-scores of height-for-age (HAZ), weight-for-age (WAZ), and weight-for-height (WHZ) associated with diarrhoea among children younger than 5 years. On Aug 8, 2016, we performed detailed literature searches using the EBSCO, Embase, and PubMed databases with no restrictions on publication date or language (appendix). We updated our search on Aug 23, 2017. We also examined the reference list of a 2015 meta-analysis^13^ for additional publications and did analyses of microdata (individual-level data) that we obtained for this study. We excluded exposures that occurred in children older than 5 years and outcomes assessed at older than 12 years (appendix). We limited our analysis to studies that could assess nutritional status in terms of continuous HAZ, WAZ, and WHZ. We excluded studies that limited these assessments to dichotomised variables, such as stunting, underweight, and wasting.

We selected these growth indicators because they are health related, are quantifiable, have sufficient literature or available microdata for meta-analyses, and are quantified in GBD 2016.^14^, ^15^ We extracted both unadjusted and adjusted effect sizes from the scientific literature and did analyses of the adjusted effect sizes. We standardised the effect sizes to represent the change per day of diarrhoea. We assumed a linear relationship between diarrhoea and growth indicators. For the microdata, we did a standard panel regression accounting for child sex, age in months, and duration between measurements. We assumed that the relationship between diarrhoea and growth did not vary by age as we had insufficient data to evaluate this. We performed random-effects meta-analyses to obtain effect sizes for the change in HAZ, WAZ, and WHZ per day of diarrhoea.

### DALYs associated with undernutrition attributable to diarrhoeal diseases

To calculate the DALYs associated with childhood undernutrition attributable to diarrhoeal diseases, we estimated the log-linear relative risk (RR) of disease and mortality for diarrhoea, lower respiratory infections, and measles per unit change in HAZ, WAZ, and WHZ on the basis of hazard ratios reported in a 2013 pooled analysis (appendix).^16^ Although hazard ratios might not be equivalent to risk ratios in many circumstances, we assume that hazard ratios can be treated similarly to risk ratios for assessment of the association between a risk and an outcome in estimation of an attributable burden.^17^ In this report, hazard ratios (HR) will be used as proxies for risk ratios and referred to as HRs. These HRs capture the relationships between undernutrition indicators and infectious disease risk. We applied the HRs to disease morbidity and mortality because of an absence of evidence evaluating disease morbidity exclusively. The HRs for stunting and wasting are adjusted to account for their covariance with each other and with underweight as described previously.^15^ We then used the effect sizes from our meta-analyses and modelled diarrhoea incidence (from GBD 2016) to calculate an outcome-specific population-attributable fraction (PAF) for the three undernutrition indicators:

$$PAF=1\frac{-}{diarrhoea\;incidence\times episode\;duration\times \frac{\Delta zscore}{day\;of\;diarrhoea}\times HR}$$

where diarrhoea incidence is the modelled number of episodes per child-year in GBD 2016 and varied by year, age, location, and sex, episode duration is the mean duration in days per diarrhoea episode duration in days per diarrhoea episode (4·3 days, 95% CI 4·2–4·4), Δzscore is the unit change per day of diarrhoea, and HR is the relative risk of each outcome per unit change in *Z*-score. The overall undernutrition PAF for each outcome was calculated as:

$$PAF_{undernutrition}=1-\left[\left(1-PAF_{WAZ}\right)\times \left(1-PAF_{HAZ}\right)\times \left(1-PAF_{WHZ}\right)\right]$$

To estimate the number of childhood undernutrition DALYs attributable to diarrhoea, we multiplied the summary PAF by the GBD 2016 DALYs for each outcome attributable to undernutrition (diarrhoea, lower respiratory infections, and measles).

### DALYs associated with PEM attributable to diarrhoeal diseases

PEM in GBD 2016 is attributed entirely to WHZ and WAZ, but not HAZ. We used the prevalence of underweight and wasting from GBD 2016, defined as less than 2 SDs below the global mean weight-for-age and weight-for-height values. We used the diarrhoeal disease incidence and effect size from the meta-analysis to estimate the counterfactual prevalence of underweight and wasting in the absence of diarrhoea, defined as:

$$Prevalence_{counterfactual}=prevalence+\frac{\Delta zscore}{day\;of\;diarrhoea}\times diarrhoea\;incidence\times episode\;duration$$

where prevalence is the prevalence of wasting or underweight, represented as a *Z*-score, Δzscore is the change in WAZ or WHZ from our meta-analysis, diarrhoea incidence is the incidence per person-year, and episode duration is the mean duration of each diarrhoeal episode (4·3 days [95% CI 4·2–4·4]). We estimated the percentage change in the cumulative density from a normal distribution:

$$PAF=\frac{prevalence_{counterfactual}-prevalence_{GBD\;2016\;estimate}}{prevalence_{GBD\;2016\;estimate}}$$

The percentage difference between the observed and counterfactual prevalence is the PAF (appendix). We calculated PAFs independently for mild, moderate, and severe undernutrition, age group, geography, sex, and year. PEM-associated DALYs attributable to diarrhoeal diseases are the PAFs multiplied by the number of DALYs due to PEM.

### Incorporation into the GBD framework

We estimated long-term DALYs due to diarrhoea for both sexes; every GBD geography; the years 1990, 1995, 2000, 2005, 2010, and 2016; and under-5 age groups. The GBD study estimates every outcome, cause, and risk at a cascade of geographies. This approach means estimation at the global, super-regional, regional, national, and sometimes subnational level. In GBD, four under-5 age groups exist. We did not estimate long-term DALYs due to diarrhoea in the neonatal age groups (0–28 days) because undernutrition in this age being due to causes other than preterm birth or other birth complications is implausible and because of the short time window for this age group. However, we consider the acute diarrhoea DALYs from each age group among children younger than 5 years, including neonates, in our analysis. We discuss percentage increase after accounting for long-term sequelae as the percentage difference between the total (acute and long-term) and acute DALYs. We assessed temporal trends by finding the relative percentage difference in DALYs per 1000 between the years 1990 and 2016. We present results for each country and for super-regions in the GBD study where the values for the super-regions are the population-weighted aggregates of the country estimates in that super-region.

We calculated uncertainty in the final DALY estimates using 1000 draws of each input parameter, including diarrhoea incidence, the effect size from the meta-analyses, the RR of each outcome, and the number of DALYs for each outcome (PEM, lower respiratory infections, diarrhoea, and measles). Our final DALY point estimate is the mean DALY estimate, with the lower uncertainty interval defined by the 2·5 percentile of the estimate and the upper uncertainty intervals defined by the 97·5 percentile of the estimate.

### Role of the funding source

The funder of the study had no role in study design, data collection, data analysis, or writing of the report. The corresponding author had full access to all the data in the study and had final responsibility for the decision to submit for publication.

## Results

We included 22 sources after reviewing reported values in the literature (17 sources) and incorporating individual-level data (five sources; appendix). From a series of random-effects meta-analyses, we found that per day of diarrhoea, height-for-age decreased by 0·0033 SDs (95% CI 0·0024–0·0041; p=4·43 × 10^−14^), weight-for-age decreased by 0·0077 SDs (0·0058–0·0097; p=3·19 × 10^−15^), and weight-for-height decreased by 0·0096 SDs (0·0067–0·0125; p=7·78 × 10^−11^; appendix).

Diarrhoeal diseases were responsible for about 40 125 700 DALYs from diarrhoea incidence and deaths among children younger than 5 years in 2016 (table), including about 446 000 deaths (390 900–504 600) and 1105 million episodes (962 million to 1275 million).^3^, ^7^, ^8^ After inclusion of the long-term sequelae associated with undernutrition, diarrhoea was responsible for 55 778 000 DALYs, an increase of nearly 40% (39·0% [33·0–46·6]; table).

**Table:** DALYs associated with diarrhoea among children younger than 5 years in 2016

		**Acute DALYs**	**Long-term sequelae DALYs**	**Total DALYs**	**Total DALYs per 1000**	**Percentage increase (%)**
**Global**		40 125 712 (35 442 905–45 159 482)	15 652 253 (12 951 273–18 806 108)	55 777 964 (49 125 416–62 396 194)	86·7 (76·4–97·0)	39·0% (33·0–46·6)
**High-income**		126 422 (104 309–152 026)	18 942 (14 631–24 241)	145 364 (120 282–174 989)	2·5 (2·1–3·0)	15·0% (12·8–17·6)
High-income North America		50 850 (43 687–58 504)	5794 (4477–7354)	56 644 (48 680–65 652)	2·6 (2·3–3·1)	11·4% (9·4–13·7)
	Canada	2186 (1668–2819)	220 (158–304)	2406 (1833–3101)	1·2 (0·9–1·6)	10·1% (8·0–12·7)
	Greenland	9 (6–16)	2 (1–3)	11 (7–18)	3·3 (2·1–5·2)	20·5% (12·6–30·2)
	USA	48 648 (41 914–55 846)	5655 (4455–7195)	54 303 (46 706–62 981)	2·8 (2·4–3·2)	11·6% (9·7–14·0)
Australasia		1471 (1169–1828)	98 (71–134)	1570 (1248–1935)	0·9 (0·7–1·1)	6·7% (5·2–8·5)
	Australia	1122 (868–1449)	68 (49–93)	1190 (926–1519)	0·8 (0·6–1·0)	6·1% (4·5–8·0)
	New Zealand	349 (262–454)	33 (23–45)	382 (287–492)	1·3 (1·0–1·7)	9·5% (7·3–12·1)
High-income Asia Pacific		7897 (6630–9347)	645 (491–843)	8542 (7154–10 081)	1·1 (0·9–1·3)	8·2% (6·7–9·8)
	Brunei	47 (35–62)	7 (5–10)	54 (41–71)	1·6 (1·2–2·0)	15·7% (10·9–21·8)
	Japan	6130 (5096–7233)	522 (399–667)	6651 (5531–7833)	1·3 (1·1–1·5)	8·5% (7·0–10·2)
	Singapore	124 (91–160)	18 (12–26)	143 (105–183)	0·8 (0·6–1·0)	14·9% (11·2–19·9)
	South Korea	1596 (1155–2160)	109 (74–156)	1705 (1244–2301)	0·8 (0·6–1·0)	6·8% (5·1–9·0)
Western Europe		42 939 (32 450–56 053)	6821 (4971–9524)	49 761 (37 676–65 321)	2·2 (1·7–2·9)	15·9% (13·6–18·6)
	Andorra	6 (4–8)	1 (1–2)	7 (5–10)	2·4 (1·7–3·3)	19·8% (14·9–28·1)
	Austria	936 (664–1275)	168 (112–246)	1103 (782–1519)	2·8 (2·0–3·9)	17·9% (15·0–21·3)
	Belgium	1632 (1201–2162)	296 (206–422)	1928 (1407–2549)	3·1 (2·3–4·1)	18·1% (15·0–21·7)
	Cyprus	174 (127–235)	27 (19–39)	202 (146–272)	3·9 (2·8–5·3)	15·6% (12·8–18·8)
	Denmark	738 (552–980)	119 (84–169)	857 (638–1150)	3·0 (2·2–4·0)	16·1% (13·3–19·2)
	Finland	706 (469–1005)	133 (82–209)	839 (551–1211)	2·9 (1·9–4·2)	18·7% (15·5–22·4)
	France	8445 (6365–11 206)	1082 (752–1522)	9527 (7162–12 635)	2·4 (1·8–3·2)	12·8% (10·4–15·5)
	Germany	7837 (5513–10 665)	1428 (954–2117)	9265 (6488–12 595)	2·7 (1·9–3·6)	18·2% (15·1–21·6)
	Greece	723 (489–1031)	167 (114–243)	890 (613–1273)	1·9 (1·3–2·7)	23·2% (18·1–30·0)
	Iceland	43 (31–59)	8 (5–11)	50 (36–70)	2·4 (1·7–3·3)	17·5% (14·6–20·8)
	Ireland	668 (479–908)	107 (74–156)	774 (556–1063)	2·1 (1·5–2·8)	16·0% (13·2–19·1)
	Israel	1391 (1063–1800)	168 (119–235)	1559 (1188–2022)	1·9 (1·4–2·4)	12·0% (9·8–14·6)
	Italy	3155 (2229–4260)	426 (291–589)	3581 (2523–4805)	1·3 (0·9–1·8)	13·5% (11·2–16·3)
	Luxembourg	75 (55–97)	11 (7–15)	86 (63–111)	3·0 (2·2–3·8)	14·0% (11·6–16·8)
	Malta	20 (15–27)	3 (2–4)	23 (17–31)	1·1 (0·8–1·5)	15·8% (12·8–19·7)
	Netherlands	2252 (1592–3067)	433 (286–642)	2685 (1890–3691)	3·1 (2·2–4·2)	19·2% (15·9–23·0)
	Norway	509 (362–681)	76 (50–108)	584 (415–785)	2·1 (1·5–2·8)	14·9% (12·4–17·8)
	Portugal	747 (567–954)	109 (79–147)	856 (657–1090)	1·9 (1·4–2·4)	14·6% (11·8–17·7)
	Spain	3280 (2374–4400)	483 (318–695)	3763 (2711–5026)	1·6 (1·1–2·1)	14·7% (12·2–17·7)
	Sweden	1137 (808–1560)	196 (133–286)	1333 (943–1843)	2·3 (1·7–3·2)	17·2% (14·5–20·4)
	Switzerland	1133 (847–1511)	175 (119–251)	1308 (973–1756)	3·2 (2·3–4·2)	15·4% (12·7–18·2)
	England	5763 (4349–7590)	1048 (788–1409)	6811 (5223–8904)	2·1 (1·6–2·8)	18·2% (15·3–21·6)
	Northern Ireland	321 (229–420)	55 (37–78)	376 (267–491)	3·2 (2·3–4·1)	17·2% (13·9–20·9)
	Scotland	633 (470–839)	85 (59–119)	718 (534–951)	2·6 (2·0–3·5)	13·4% (11·0–16·1)
	Wales	584 (434–770)	113 (79–159)	697 (519–916)	4·3 (3·2–5·7)	19·4% (16·2–23·0)
Southern Latin America		23 264 (18 492–28 974)	8131 (6115–10 817)	31 395 (25 222–39 226)	6·3 (5·0–7·8)	35·1% (27·9–43·8)
	Argentina	19 632 (15 554–24 554)	7523 (5609–10 170)	27 154 (21 802–34 164)	7·7 (6·1–9·6)	38·5% (30·3–48·9)
	Chile	2768 (2017–3692)	698 (488–955)	3466 (2554–4563)	2·8 (2·1–3·7)	25·4% (19·2–33·3)
	Uruguay	864 (589–1258)	181 (109–284)	1045 (709–1518)	4·4 (3·0–6·4)	21·0% (15·4–27·8)
**Central Europe, eastern Europe, and central Asia**		263 910 (206 547–332 928)	284 432 (205 395–382 154)	548 342 (428 540–685 480)	18·0 (14·1–22·5)	108·4% (81·4–143·1)
Eastern Europe		60 595 (44 006–79 621)	38 502 (29 757–48 593)	99 097 (76 745–124 688)	7·2 (5·6–9·1)	64·3% (49·7–82·3)
	Belarus	2045 (1382–2889)	748 (493–1067)	2793 (1902–3925)	4·7 (3·2–6·6)	36·9% (28·3–48·6)
	Estonia	474 (320–663)	251 (174–349)	725 (507–1000)	9·4 (6·6–13·0)	53·3% (43·8–65·5)
	Latvia	678 (457–944)	324 (219–460)	1002 (685–1384)	7·8 (5·3–10·7)	48·1% (38·3–61·0)
	Lithuania	1085 (737–1514)	584 (407–796)	1669 (1166–2267)	9·5 (6·6–12·8)	54·3% (43·8–66·2)
	Moldova	1134 (791–1539)	884 (569–1336)	2018 (1420–2747)	9·1 (6·4–12·3)	78·3% (56·7–106·9)
	Russia	43 308 (31 759–56 623)	28 671 (21 769–36 447)	71 979 (56 085–90 229)	7·3 (5·7–9·1)	67·0% (50·6–88·0)
	Ukraine	11 871 (8257–16 284)	6739 (4213–10 670)	18 610 (12 887–25 857)	7·2 (5·0–9·9)	56·9% (41·3–80·5)
Central Europe		32 928 (23 638–43 862)	23 569 (17 940–30 516)	56 497 (43 020–72 983)	9·9 (7·5–12·8)	72·4% (56·8–91·1)
	Albania	787 (544–1068)	1301 (767–2120)	2087 (1411–3109)	12·0 (8·1–17·9)	167·3% (104·5–264·9)
	Bosnia and Herzegovina	812 (599–1073)	291 (200–418)	1104 (810–1462)	6·4 (4·7–8·5)	35·9% (28·7–43·9)
	Bulgaria	2008 (1416–2739)	1837 (1151–2877)	3846 (2666–5485)	11·3 (7·8–16·1)	91·7% (64·3–130·9)
	Croatia	1113 (774–1505)	394 (271–545)	1507 (1049–2028)	7·6 (5·3–10·2)	35·6% (29·7–42·6)
	Czech Republic	2845 (1967–3924)	909 (639–1274)	3754 (2623–5159)	6·6 (4·6–9·1)	32·1% (26·4–39·2)
	Hungary	2351 (1654–3203)	919 (609–1330)	3270 (2317–4456)	6·4 (4·6–8·8)	39·3% (30·6–50·8)
	Macedonia	661 (477–902)	388 (251–582)	1049 (751–1466)	9·2 (6·6–12·9)	58·8% (43·6–79·6)
	Montenegro	108 (75–146)	47 (30–67)	155 (108–209)	4·2 (2·9–5·7)	43·5% (31·4–60·3)
	Poland	10 258 (6810–14 380)	4307 (2806–6274)	14 565 (9797–20 607)	7·3 (4·9–10·4)	42·2% (33·3–53·3)
	Romania	7365 (5574–9554)	10 986 (8000–14 926)	18 351 (14 185–23 623)	23·0 (17·8–29·6)	150·4% (111·5–194·8)
	Serbia	2198 (1583–2897)	1142 (860–1477)	3340 (2497–4265)	8·1 (6·1–10·4)	52·5% (41·8–67·0)
	Slovakia	1816 (1253–2492)	1106 (753–1564)	2922 (2078–3929)	9·8 (7·0–13·2)	61·3% (46·7–81·7)
	Slovenia	605 (396–844)	211 (134–316)	816 (534–1143)	7·7 (5·0–10·8)	34·8% (28·4–42·1)
Central Asia		170 387 (125 198–234 301)	190 380 (133 429–266 692)	360 767 (268 785–476 224)	32·8 (24·4–43·2)	113·2% (79·6–155·5)
	Armenia	1407 (1005–1880)	1870 (1261–2707)	3277 (2318–4541)	14·6 (10·3–20·2)	133·5% (96·0–181·3)
	Azerbaijan	18 732 (11 311–30 533)	31 683 (18 738–50 112)	50 415 (30 110–80 054)	54·4 (32·5–86·4)	171·8% (120·5–240·4)
	Georgia	1801 (1281–2467)	976 (650–1417)	2777 (1990–3761)	9·9 (7·1–13·5)	54·4% (40·2–73·5)
	Kazakhstan	9155 (6463–12 641)	6789 (4328–10 170)	15 945 (11 130–22 152)	8·2 (5·7–11·3)	74·4% (52·7–101·4)
	Kyrgyzstan	17 909 (13 573–23 708)	13 853 (10 039–18 373)	31 761 (24 145–40 873)	39·2 (29·8–50·4)	77·9% (58·2–100·2)
	Mongolia	1250 (867–1704)	4893 (3080–7291)	6143 (4224–8750)	16·5 (11·4–23·5)	399·0% (239·3–614·4)
	Tajikistan	91 233 (50 109–154 336)	72 358 (41 809–117 541)	163 591 (95 481–261 019)	149·0 (86·9–237·7)	80·9% (56·6–112·5)
	Turkmenistan	11 694 (7553–18 613)	22 894 (14 205–34 727)	34 588 (22 041–51 970)	64·5 (41·1–97·0)	197·8% (144·6–269·7)
	Uzbekistan	17 206 (10 801–26 065)	52 686 (26 047–93 836)	69 892 (37 608–116 929)	14·5 (7·8–24·3)	303·9% (202·9–432·3)
**Latin America and the Caribbean**		1004 324 (864 309–1177 403)	700 024 (589 718–819 360)	1704 348 (1482 667–1956 000)	36·0 (31·4–41·4)	69·9% (59·6–80·9)
Central Latin America		378 280 (331 484–429 640)	180 640 (149 052–217 570)	558 920 (493 576–636 880)	25·0 (22·1–28·5)	47·8% (40·0–56·2)
	Colombia	29 855 (22 547–37 937)	17 491 (12 596–23 468)	47 346 (35 772–60 630)	14·6 (11·1–18·7)	58·8% (45·4–74·0)
	Costa Rica	2311 (1606–3180)	932 (574–1423)	3243 (2214–4551)	10·0 (6·8–14·0)	40·2% (31·6–51·5)
	El Salvador	8227 (5647–12 323)	4432 (2882–6810)	12 659 (8667–18 710)	27·0 (18·5–39·9)	54·2% (41·0–70·3)
	Guatemala	94 791 (75 446–117 097)	86 478 (70 493–107 275)	181 270 (148 517–222 264)	94·7 (77·6–116·1)	91·8% (73·8–111·9)
	Honduras	27 422 (18 714–38 562)	10 993 (7514–15 505)	38 415 (26 784–53 040)	41·0 (28·6–56·6)	40·4% (31·1–52·2)
	Mexico	140 290 (122 438–162 392)	42 000 (33 867–51 763)	182 290 (158 942–211 028)	15·6 (13·6–18·1)	30·0% (25·0–35·7)
	Nicaragua	12 326 (8962–16 573)	6759 (5059–8904)	19 085 (14 522–24 563)	31·3 (23·8–40·3)	55·5% (41·5–74·2)
	Panama	8795 (6318–11 928)	5678 (3823–8348)	14 473 (10 158–19 792)	39·8 (27·9–54·4)	64·6% (51·5–80·4)
	Venezuela	54 263 (41 520–69 687)	31 046 (23 455–40 782)	85 309 (66 010–108 458)	30·1 (23·3–38·3)	57·4% (47·0–70·7)
Andean Latin America		90 363 (72 676–111 295)	99 578 (78 133–126 364)	189 941 (155 282–229 631)	29·0 (23·7–35·0)	110·9% (87·4–139·1)
	Bolivia	33 409 (22 054–49 467)	48 976 (33 198–70 483)	82 385 (58 177–114 936)	61·7 (43·6–86·1)	150·1% (100·7–212·8)
	Ecuador	21 016 (15 746–27 254)	21 020 (15 067–28 294)	42 036 (31 603–54 074)	24·9 (18·7–32·0)	100·5% (78·0–128·9)
	Peru	35 938 (27 269–48 053)	35 007 (25 238–47 401)	70 946 (54 387–91 993)	20·1 (15·4–26·0)	98·3% (71·2–131·0)
The Caribbean		254 822 (163 038–400 376)	119 329 (77 040–178 409)	374 151 (244 001–581 746)	90·7 (59·1–141·0)	47·3% (36·3–60·3)
	Antigua and Barbuda	47 (33–65)	30 (19–46)	76 (53–108)	16·5 (11·5–23·3)	62·9% (49·0–80·2)
	The Bahamas	227 (144–357)	160 (77–317)	387 (225–670)	12·1 (7·1–21·0)	69·2% (47·2–98·6)
	Barbados	94 (61–142)	69 (35–125)	163 (98–264)	10·4 (6·3–16·8)	71·4% (49·9–100·5)
	Belize	531 (290–932)	338 (158–665)	868 (449–1551)	21·0 (10·9–37·5)	62·7% (46·2–82·2)
	Bermuda	19 (14–27)	7 (5–10)	27 (18–37)	6·0 (4·2–8·3)	36·3% (28·4–46·8)
	Cuba	2354 (1788–3024)	795 (593–1045)	3148 (2442–3959)	5·1 (4·0–6·4)	33·9% (27·0–42·6)
	Dominica	49 (32–70)	31 (18–49)	79 (51–117)	14·7 (9·4–21·7)	62·7% (46·9–82·0)
	Dominican Republic	22 370 (15 683–32 175)	13 369 (8851–19 516)	35 739 (25 130–50 917)	35·1 (24·7–50·0)	60·2% (44·6–78·2)
	Grenada	71 (48–103)	63 (35–111)	134 (84–208)	11·3 (7·1–17·4)	88·8% (63·8–123·1)
	Guyana	1742 (1140–2465)	703 (455–1007)	2445 (1614–3432)	34·3 (22·6–48·1)	40·7% (31·7–51·5)
	Haiti	220 985 (130 989–366 349)	124 316 (74 180–198 837)	345 302 (209 891–567 416)	228·3 (138·8–375·2)	56·8% (43·3–73·5)
	Jamaica	2497 (1502–4251)	1293 (697–2240)	3790 (2273–6292)	14·2 (8·5–23·5)	51·9% (37·8–69·7)
	Puerto Rico	1040 (785–1359)	340 (242–466)	1381 (1056–1810)	5·7 (4·4–7·5)	32·8% (26·3–40·4)
	Saint Lucia	94 (61–138)	65 (36–111)	159 (99–249)	15·9 (9·9–24·8)	68·3% (50·5–90·4)
	Saint Vincent and the Grenadines	104 (67–158)	58 (35–98)	162 (102–251)	17·0 (10·7–26·3)	56·2% (42·9–71·0)
	Suriname	1564 (884–2588)	837 (449–1419)	2401 (1377–3978)	45·5 (26·1–75·4)	54·1% (39·9–72·5)
	Trinidad and Tobago	500 (271–934)	193 (81–416)	694 (353–1332)	7·8 (4·0–15·0)	37·7% (27·7–50·9)
	Virgin Islands	26 (18–35)	15 (10–22)	41 (29–55)	7·5 (5·4–10·1)	59·9% (41·7–88·6)
Tropical Latin America		273 592 (228 092–324 228)	271 083 (227 613–318 028)	544 675 (460 867–631 756)	38·2 (32·3–44·3)	99·4% (86·6–113·6)
	Brazil	261 422 (217 495–310 145)	257 810 (214 808–303 438)	519 232 (440 641–605 417)	37·2 (31·6–43·4)	98·9% (86·4–113·5)
	Paraguay	12 170 (9046–15 901)	13 189 (9473–18 066)	25 359 (19 071–33 244)	81·1 (61·0–106·3)	108·9% (86·5–139·9)
**Southeast Asia, east Asia, and Oceania**		1555 717 (1338 820–1827 359)	636 042 (500 662–804 298)	2191 760 (1893 782–2543 380)	17·4 (15·0–20·1)	41·0% (32·1–51·8)
East Asia		234 944 (190 755–291 799)	73 671 (56 937–94 409)	308 615 (255 625–377 587)	4·7 (3·9–5·7)	31·5% (24·1–40·6)
	China	167 323 (140 598–201 545)	52 510 (40 674–66 981)	219 833 (185 311–264 092)	3·6 (3·0–4·3)	31·5% (24·4–40·0)
	North Korea	64 813 (37 824–109 764)	38 662 (22 921–63 483)	103 475 (61 890–163 185)	31·5 (18·8–49·7)	61·2% (39·0–89·5)
	Taiwan	2808 (2025–3795)	640 (440–905)	3448 (2484–4618)	3·3 (2·4–4·4)	22·8% (18·2–28·2)
Southeast Asia		1279 482 (1089 080–1510 635)	680 378 (541 346–859 868)	1959 861 (1672 444–2280 419)	33·3 (28·5–38·8)	53·3% (43·1–66·6)
	Cambodia	34 352 (23 085–50 531)	58 038 (40 257–79 291)	92 390 (66 630–126 727)	50·4 (36·4–69·2)	172·9% (115·5–244·0)
	Indonesia	724 943 (604 164–877 963)	349 529 (269 818–458 335)	1074 473 (903 171–1296 756)	44·7 (37·6–53·9)	48·4% (38·2–63·1)
	Laos	75 364 (33 971–138 963)	70 406 (34 335–127 694)	145 770 (70 464–261 186)	135·1 (65·3–242·0)	95·8% (62·0–142·2)
	Malaysia	10 660 (7827–14 330)	3959 (2702–5714)	14 619 (10 882–19 422)	5·6 (4·2–7·5)	37·3% (27·4–51·2)
	Maldives	102 (74–138)	28 (19–41)	130 (96–176)	3·5 (2·6–4·8)	27·7% (20·3–36·8)
	Mauritius	492 (375–635)	171 (120–245)	663 (506–874)	10·0 (7·6–13·2)	34·8% (27·2–44·4)
	Myanmar	113 676 (63 868–192 713)	80 856 (45 407–141 135)	194 533 (112 624–326 598)	31·1 (18·0–52·2)	72·5% (49·2–103·0)
	Philippines	255 599 (172 580–381 272)	126 251 (92 614–166 232)	381 850 (271 926–530 156)	33·0 (23·5–45·8)	50·6% (34·5–68·0)
	Sri Lanka	4783 (3324–6501)	1805 (1143–2651)	6588 (4577–8998)	4·7 (3·3–6·5)	37·8% (28·9–50·1)
	Seychelles	30 (22–40)	24 (17–35)	54 (40–73)	6·5 (4·7–8·7)	80·0% (59·5–105·2)
	Thailand	13 863 (10 007–18 556)	4897 (3276–6959)	18 760 (13 473–24 962)	7·5 (5·4–10·0)	35·4% (27·3–44·1)
	Timor-Leste	18 203 (9792–30 758)	8553 (4743–13 826)	26 756 (14 900–44 177)	162·7 (90·6–268·7)	47·8% (35·1–64·3)
	Vietnam	27 227 (18 845–36 638)	27 948 (18 088–40 915)	55 175 (38 680–74 795)	7·7 (5·4–10·5)	103·7% (71·5–144·7)
Oceania		40 023 (23 426–66 397)	47 221 (25 547–81 688)	87 244 (51 002–141 375)	63·2 (36·9–102·4)	119·3% (80·4–169·8)
	American Samoa	32 (23–42)	20 (13–27)	51 (38–68)	6·9 (5·0–9·1)	61·7% (46·5–81·1)
	Federated States of Micronesia	50 (32–76)	48 (25–83)	98 (60–156)	10·0 (6·0–15·8)	96·3% (64·5–139·9)
	Fiji	1345 (716–2386)	1047 (565–1804)	2393 (1334–4082)	43·9 (24·5–74·9)	79·3% (53·8–112·2)
	Guam	56 (39–77)	68 (43–100)	124 (89–167)	7·1 (5·1–9·6)	122·4% (81·8–177·8)
	Kiribati	639 (309–1191)	497 (238–916)	1136 (561–2049)	83·6 (41·2–150·7)	79·7% (52·6–116·7)
	Marshall Islands	66 (39–110)	85 (41–161)	150 (83–264)	15·3 (8·4–26·8)	128·6% (81·6–190·6)
	Northern Mariana Islands	58 (39–84)	26 (16–42)	84 (56–125)	5·4 (3·6–8·1)	45·1% (33·1–62·0)
	Papua New Guinea	34 511 (18 126–59 686)	43 360 (22 470–77 261)	77 871 (41 981–130 724)	74·8 (40·3–125·5)	128·0% (81·7–189·0)
	Samoa	110 (73–159)	120 (59–232)	230 (137–378)	8·3 (5·0–13·7)	107·9% (65·4–177·1)
	Solomon Islands	2052 (1210–3233)	1289 (745–2085)	3341 (2009–5115)	40·8 (24·5–62·4)	63·7% (44·5–87·2)
	Tonga	85 (52–132)	81 (45–139)	167 (102–272)	12·2 (7·4–19·9)	95·4% (67·5–135·3)
	Vanuatu	860 (485–1478)	908 (465–1674)	1768 (958–3100)	43·8 (23·7–76·8)	106·2% (70·3–152·0)
**North Africa and the Middle East**		2544 397 (1971 087–3264 069)	1204 970 (904 070–1576 142)	3749 367 (2942 873–4751 247)	59·0 (46·3–74·8)	47·6% (38·4–59·3)
	Afghanistan	397 941 (235 409–616 694)	424 285 (254 103–653 576)	822 225 (510 497–1228 901)	173·4 (107·7–259·2)	108·7% (75·1–152·7)
	Algeria	32 632 (22 492–47 409)	15 381 (9569–24 233)	48 013 (32 306–70 653)	9·4 (6·3–13·9)	47·4% (33·4–66·5)
	Bahrain	357 (252–489)	205 (139–290)	562 (403–764)	5·8 (4·2–7·9)	57·7% (45·0–74·1)
	Egypt	829 830 (504 593–1327 052)	311 983 (202 659–472 688)	1141 813 (718 003–1778 764)	95·5 (60·0–148·8)	38·1% (29·8–48·3)
	Iran	63 589 (39 324–103 647)	36 965 (18 466–71 067)	100 554 (59 085–172 413)	14·2 (8·3–24·3)	57·2% (42·1–78·8)
	Iraq	176 068 (92 019–278 225)	98 412 (47 330–159 563)	274 480 (141 552–422 605)	37·1 (19·1–57·1)	56·2% (40·9–76·8)
	Jordan	4853 (3551–6377)	3545 (2338–4986)	8398 (6163–10 804)	8·7 (6·4–11·3)	73·7% (51·1–101·9)
	Kuwait	1276 (913–1729)	589 (396–861)	1865 (1324–2554)	2·8 (2·0–3·8)	46·4% (35·8–60·5)
	Lebanon	2139 (1463–2938)	789 (503–1164)	2928 (2011–4014)	9·7 (6·7–13·3)	36·9% (29·0–47·0)
	Libya	3650 (2479–5212)	1548 (970–2406)	5198 (3473–7538)	10·6 (7·1–15·3)	42·3% (33·0–53·9)
	Morocco	87 117 (55 718–132 357)	26 987 (17 651–39 291)	114 104 (74 429–169 969)	50·8 (33·2–75·7)	31·3% (24·5–39·7)
	Palestine	6971 (4831–9748)	3447 (1976–5593)	10 417 (6949–15 040)	9·7 (6·5–14·1)	49·2% (34·4–70·2)
	Oman	1809 (1268–2475)	799 (554–1135)	2608 (1884–3518)	6·1 (4·4–8·2)	44·5% (33·6–58·9)
	Qatar	358 (253–495)	107 (70–163)	465 (332–638)	3·7 (2·6–5·0)	30·1% (22·4–40·4)
	Saudi Arabia	9235 (6950–11 939)	2011 (1416–2805)	11 246 (8368–14 611)	4·4 (3·3–5·7)	21·8% (18·0–26·0)
	Sudan	316 837 (133 660–638 048)	220 655 (111 171–404 196)	537 492 (254 321–1020 827)	145·6 (68·9–276·5)	74·1% (46·4–119·6)
	Syria	7426 (5196–10 208)	5735 (3560–9024)	13 161 (9001–18 680)	6·8 (4·6–9·6)	77·4% (55·6–108·3)
	Tunisia	4339 (3149–5811)	1761 (1173–2507)	6100 (4460–8159)	6·6 (4·8–8·8)	40·7% (31·3–53·4)
	Turkey	39 155 (28 393–54 115)	16 885 (10 601–25 649)	56 040 (40 025–77 262)	9·0 (6·4–12·4)	43·1% (31·6–57·0)
	United Arab Emirates	3360 (2316–4685)	1343 (841–2046)	4703 (3196–6719)	5·1 (3·4–7·2)	40·0% (31·3–51·8)
	Yemen	553 420 (228 047–945 916)	261 525 (112 953–445 330)	814 945 (341 307–1349 376)	180·4 (75·6–298·7)	47·9% (37·1–62·4)
**South Asia**		9134 318 (7755 473–10 851 268)	3278 190 (2705 240–3960 683)	12 412 508 (10 723 546–14 408 198)	77·7 (67·1–90·2)	36·1% (29·2–44·0)
	Bangladesh	305 736 (202 406–430 192)	178 872 (127 264–246 408)	484 609 (346 605–637 389)	32·5 (23·2–42·7)	60·4% (38·3–92·0)
	Bhutan	1976 (1237–2996)	2512 (1759–3389)	4489 (3272–6000)	59·4 (43·3–79·3)	131·8% (84·2–206·7)
	India	5912 144 (5024 503–6980 262)	2111 276 (1720 151–2591 184)	8023 420 (6934 539–9287 808)	69·1 (59·7–80·0)	35·9% (28·6–43·9)
	Nepal	94 203 (56 391–153 775)	67 365 (46 076–94 671)	161 568 (109 045–243 714)	38·4 (25·9–57·9)	74·8% (47·5–115·1)
	Pakistan	2820 258 (1956 738–3818 966)	972 455 (714 556–1285 862)	3792 713 (2747 962–5101 313)	155·2 (112·4–208·7)	34·9% (27·7–44·3)
**Sub-Saharan Africa**		25 496 626 (21 499 324–29 988 405)	11 223 063 (9098 125–13 701 147)	36 719 688 (31 116 143–42 803 762)	232·4 (197·0–271·0)	44·1% (36·4–53·7)
Southern sub-Saharan Africa		910 947 (739 134–1111 939)	342 971 (269 589–434 045)	1253 918 (1033 037–1515 752)	138·4 (114·0–167·2)	37·8% (30·5–46·4)
	Botswana	15 445 (8358–25 762)	2704 (1496–4520)	18 149 (9949–30 375)	66·7 (36·6–111·6)	17·7% (13·7–23·2)
	Lesotho	78 544 (53 335–112 774)	25 550 (17 300–37 072)	104 094 (72 253–147 729)	412·3 (286·2–585·1)	32·8% (25·5–41·9)
	Namibia	50 847 (29 936–77 942)	15 976 (9394–25 279)	66 822 (40 070–101 468)	196·2 (117·6–297·9)	31·8% (23·9–41·5)
	South Africa	245 513 (177 965–330 141)	123 849 (84 879–168 370)	369 362 (266 516–489 946)	68·3 (49·3–90·7)	50·5% (41·4–61·3)
	Swaziland	46 516 (29 602–66 411)	16 222 (10 792–22 929)	62 739 (42 753–87 985)	306·9 (209·1–430·4)	35·4% (26·5–48·0)
	Zimbabwe	474 082 (328 742–648 101)	165 191 (123 261–222 015)	639 273 (466 425–855 538)	246·9 (180·2–330·5)	35·3% (26·6–46·4)
Western sub-Saharan Africa		15 412 421 (12 202 248–19 099 241)	5514 808 (4311 612–6973 720)	20 927 230 (16 853 517–25 445 048)	320·5 (258·1–389·7)	35·9% (29·8–43·9)
	Benin	480 644 (324 417–663 789)	131 657 (94 703–180 034)	612 300 (427 597–822 043)	313·5 (218·9–420·8)	27·7% (20·9–36·2)
	Burkina Faso	408 365 (252 480–631 102)	234 423 (149 493–336 357)	642 788 (413 416–952 201)	199·7 (128·4–295·8)	58·6% (41·5–83·1)
	Cameroon	435 618 (265 855–674 394)	270 653 (183 093–391 021)	706 271 (478 470–1010 687)	179·6 (121·7–257·0)	63·9% (42·5–92·1)
	Cape Verde	941 (655–1331)	363 (255–494)	1304 (952–1760)	17·5 (12·8–23·7)	39·3% (27·7–54·4)
	Chad	1142 252 (797 310–1564 356)	634 554 (470 377–823 845)	1776 806 (1322 699–2297 013)	689·1 (513·0–890·9)	56·5% (41·9–77·8)
	Côte d'Ivoire	864 858 (536 377–1298 185)	306 111 (204 717–436 691)	1170 969 (765 377–1686 428)	334·9 (218·9–482·3)	36·0% (27·0–49·1)
	The Gambia	37 025 (25 534–52 515)	16 840 (12 290–22 412)	53 865 (39 227–73 033)	146·7 (106·8–198·9)	46·3% (34·3–63·5)
	Ghana	138 351 (90 157–205 439)	79 725 (53 863–113 586)	218 075 (150 551–307 629)	47·2 (32·6–66·6)	58·8% (40·5–82·1)
	Guinea	231 931 (140 481–367 723)	162 026 (115 051–221 365)	393 957 (272 220–568 095)	196·8 (136·0–283·8)	72·8% (46·6–111·3)
	Guinea-Bissau	44 111 (27 955–63 504)	17 194 (11 929–22 955)	61 304 (42 467–85 653)	200·6 (139·0–280·3)	39·9% (28·3–58·2)
	Liberia	187 407 (124 877–258 104)	62 491 (45 123–83 740)	249 898 (174 220–336 850)	352·0 (245·4–474·5)	33·8% (26·3–44·3)
	Mali	986 099 (653 739–1430 018)	300 109 (211 039–424 978)	1286 209 (879 347–1806 820)	417·5 (285·4–586·5)	30·8% (23·7–40·1)
	Mauritania	51 921 (36 534–72 690)	20 550 (15 012–26 599)	72 471 (53 167–97 953)	145·2 (106·5–196·3)	40·3% (29·7–55·1)
	Niger	1198 125 (752 031–1771 998)	441 522 (306 922–624 441)	1639 646 (1086 767–2370 464)	442·1 (293·0–639·1)	37·5% (28·4–51·0)
	Nigeria	8477 226 (5889 625–11 597 962)	2664 657 (1906 075–3587 026)	11 141 883 (7901 947–15 027 306)	371·0 (263·2–500·4)	31·7% (24·9–40·8)
	São Tomé and Príncipe	1648 (927–2668)	856 (597–1203)	2504 (1630–3780)	72·5 (47·2–109·4)	55·0% (34·4–83·9)
	Senegal	309 673 (218 642–427 650)	128 515 (95 929–166 993)	438 187 (326 035–587 284)	171·0 (127·2–229·1)	42·1% (32·0–55·1)
	Sierra Leone	325 808 (211 574–472 889)	118 709 (83 873–160 419)	444 517 (300 765–612 675)	427·1 (289·0–588·7)	37·1% (27·5–49·2)
	Togo	90 418 (56 474–137 312)	39 692 (26 978–55 929)	130 111 (86 383–187 942)	119·1 (79·1–172·0)	45·0% (32·3–63·6)
Eastern sub-Saharan Africa		6470 572 (5411 646–7543 186)	3679 486 (2969 669–4543 519)	10 150 058 (8656 954–11 708 664)	162·7 (138·8–187·7)	57·1% (45·8–71·0)
	Burundi	662 796 (437 758–978 159)	268 716 (176 921–392 470)	931 512 (632 294–1357 044)	440·4 (298·9–641·5)	41·0% (31·4–52·0)
	Comoros	6240 (3542–10 938)	4714 (2876–7257)	10 955 (6831–17 360)	112·7 (70·3–178·6)	78·6% (49·2–121·5)
	Djibouti	7184 (3696–12 760)	6269 (3705–9854)	13 454 (7864–21 405)	107·3 (62·7–170·8)	92·4% (52·3–148·9)
	Eritrea	108 583 (67 687–167 071)	49 388 (33 054–69 017)	157 971 (103 402–228 958)	210·5 (137·8–305·1)	46·6% (32·9–65·9)
	Ethiopia	1344 002 (925 183–1818 464)	728 197 (521 862–934 530)	2072 199 (1476 091–2670 266)	140·3 (99·9–180·8)	55·0% (41·0–73·1)
	Kenya	566 428 (449 052–715 006)	269 469 (218 506–334 375)	835 898 (684 542–1003 024)	126·9 (103·9–152·3)	48·2% (35·4–63·9)
	Madagascar	1013 664 (611 099–1551 095)	466 097 (320 535–641 189)	1479 761 (979 737–2145 279)	383·1 (253·6–555·4)	47·3% (33·4–67·8)
	Malawi	336 990 (221 248–492 405)	223 340 (151 604–312 425)	560 330 (395 572–785 660)	173·5 (122·5–243·2)	67·6% (48·7–96·6)
	Mozambique	363 865 (235 158–529 586)	190 778 (132 286–268 063)	554 642 (387 040–777 413)	110·5 (77·1–154·8)	53·6% (37·4–77·1)
	Rwanda	139 945 (92 328–207 843)	89 364 (61 799–124 586)	229 309 (161 300–318 421)	123·6 (86·9–171·6)	65·3% (45·6–93·1)
	Somalia	174 455 (96 543–303 397)	152 360 (94 239–221 624)	326 814 (205 457–507 363)	276·4 (173·7–429·0)	91·4% (55·6–139·4)
	South Sudan	310 000 (158 136–538 975)	414 346 (278 321–589 423)	724 346 (475 489–1057 324)	243·0 (159·5–354·7)	143·5% (78·7–237·7)
	Tanzania	525 188 (341 122–764 776)	318 177 (213 964–438 234)	843 365 (593 403–1150 829)	89·8 (63·2–122·6)	61·9% (41·7–88·3)
	Uganda	641 673 (449 078–897 394)	402 987 (287 219–550 955)	1044 660 (766 731–1382 415)	138·4 (101·6–183·2)	64·0% (44·7–89·6)
	Zambia	269 423 (178 716–391 691)	186 294 (131 419–250 077)	455 716 (323 140–631 233)	161·5 (114·5–223·6)	70·6% (50·0–96·6)
Central sub-Saharan Africa		2699 356 (1862 779–3849 054)	1737 339 (1125 465–2570 235)	4436 695 (3090 557–6236 852)	209·0 (145·6–293·8)	64·7% (49·6–85·1)
	Angola	699 114 (407 541–1100 511)	354 565 (220 329–544 268)	1053 679 (644 476–1604 526)	210·6 (128·8–320·8)	51·4% (38·5–68·3)
	Central African Republic	248 959 (154 864–383 261)	199 327 (130 425–292 010)	448 286 (302 174–655 152)	566·7 (382·0–828·2)	81·9% (56·6–116·8)
	Congo	60 582 (35 574–97 971)	36 842 (22 535–58 126)	97 423 (60 231–151 612)	130·7 (80·8–203·5)	62·0% (44·0–86·8)
	Democratic Republic of the Congo	1678 409 (943 468–2723 332)	1159 365 (640 511–1891 685)	2837 774 (1615 506–4425 438)	197·8 (112·6–308·5)	70·0% (49·6–97·7)
	Equatorial Guinea	1503 (920–2439)	2360 (1326–3983)	3862 (2385–6211)	39·4 (24·3–63·3)	160·7% (94·6–254·2)
	Gabon	10 790 (6272–17 022)	6991 (4118–10 489)	17 782 (11 075–26 456)	71·6 (44·6–106·5)	66·5% (43·6–99·1)

Data in parentheses are 95% uncertainty intervals. DALY=disability-adjusted life-year. Acute DALYs are the burden associated with immediate health loss due to diarrhoea, long-term sequelae DALYs are the burden associated with growth impairment due to diarrhoea, and total DALYs are the sum of acute and long-term DALYs.

**Figure 1:**
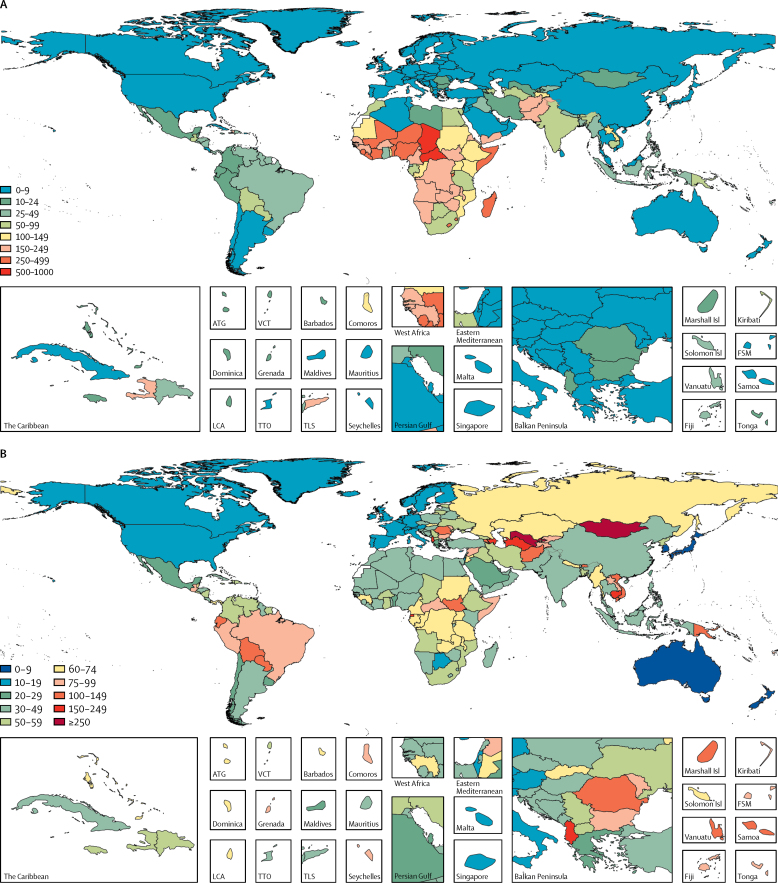
Diarrhoea DALYs The total diarrhoea DALYs represents the sum of acute and long-term sequelae DALYs per 1000 children younger than 5 years in 2016. (A) The sum of the acute DALYs (diarrhoea incidence and mortality) and long-term sequelae diarrhoea DALYs due to growth impairment per 1000 children younger than 5 years in 2016. (B) The relative percentage increase in the total number of DALYs due to diarrhoeal diseases in 2016 among children younger than 5 years after inclusion of the long-term sequelae diarrhoea DALYs compared with the acute diarrhoea DALYs only. ATG=Antigua and Barbuda. BRB=Barbados. COM=Comoros. DALYs=disability-adjusted life-years. DMA=Dominica. FJI=Fiji. FSM=Federated States of Micronesia. GRD=Grenada. KIR=Kiribati. LCA=Saint Lucia. MDV=Maldives. MHL=Marshall Islands. MLT=Malta. MUS=Mauritius. SGP=Singapore. SLB=Solomon Islands. SYC=Seychelles. TLS=Timor-Leste. TON=Tonga. TTO=Trinidad and Tobago. VCT=Saint Vincent and the Grenadines. VUT=Vanuatu. WSM=Samoa.

The total burden of diarrhoea, represented as DALYs per 1000 people, is shown in figure 1. The highest rates of diarrhoea DALYs were in sub-Saharan Africa, south Asia, and southeast Asia. After accounting for long-term sequelae associated with diarrhoea, the number of diarrhoea DALYs more than doubled in some countries (table, figure 1B, appendix). Various countries in central Asia, such as Mongolia and Uzbekistan, with low acute diarrhoea burden, increased by 300% after inclusion of long-term sequelae DALYs (table). DALYs associated with diarrhoea increased by more than two-thirds in Latin America and the Caribbean, with larger increases in Andean Latin America and lower increases in Central Latin America. The southeast Asia region increased by more than half overall, with the largest increase in Cambodia and the lowest in the Maldives.

**Figure 2:**
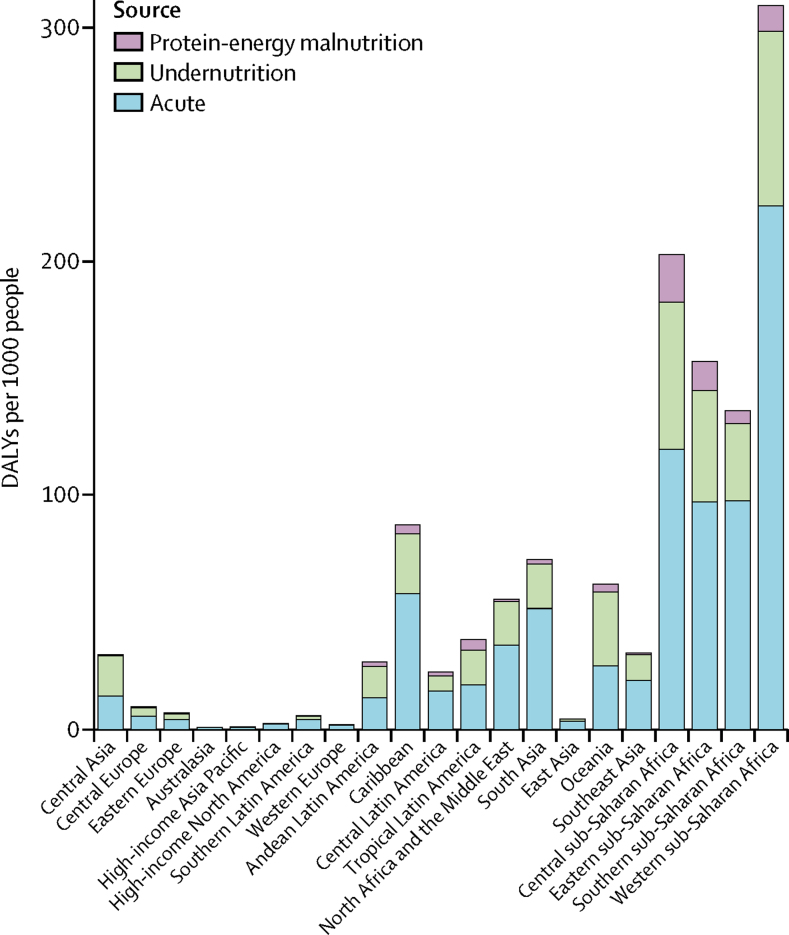
Number of short-term (acute) and long-term sequelae DALYs per 1000 children under 5 years old by Global Burden of Disease region in 2016 DALY=disability-adjusted life-year.

The total diarrhoea DALYs per 1000 children younger than 5 years occurred in western (320·5 per 1000, 95% UI 258·1–389·7) and central (209·0 per 1000, 145·6–293·8 per 1000) sub-Saharan Africa, and the lowest rate occurred in Australasia (0·9 per 1000, 0·7–1·1; figure 2). The highest rate of diarrhoeal DALYs occurred in the central Sahel region of sub-Saharan Africa, including Chad (689·1 per 1000 [95% UI 513·0–890·9]), the Central African Republic (566·7 per 1000 [382·0–828·2]), and Niger (442·1 per 1000 [293·0–639·1]; appendix; table). Acute DALYs were correlated with long-term DALYs in most locations. The total rate of diarrhoea DALYs in south Asia was 77·7 per 1000 (67·1–90·2), including 69·1 per 1000 (59·7–80·0) in India and 155·2 per 1000 (112·4–208·7) in Pakistan where the burden was predominantly due to the acute DALYs. The rate of diarrhoea DALYs varied substantially among countries in north Africa and the Middle East, ranging from 2·8 per 1000 (2·0–3·8) in Kuwait to 173·4 per 1000 (107·7–259·2) in Afghanistan. In central Europe, eastern Europe, and central Asia, rates ranged from 4·2 per 1000 (2·9–5·7) in Montenegro to 149·0 per 1000 (86·9–237·7) in Tajikistan.

**Figure 3:**
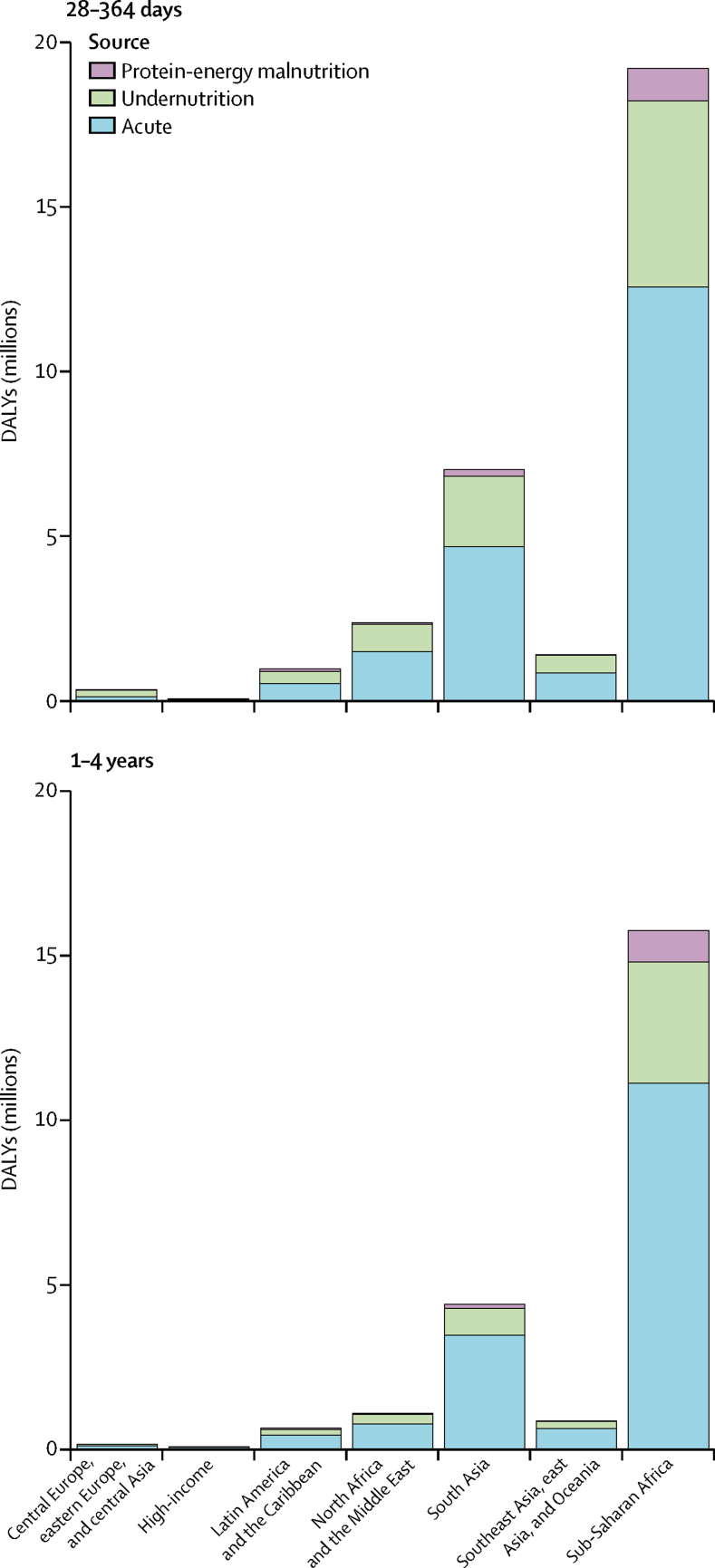
Absolute number of short-term (acute) and long-term sequelae DALYs by Global Burden of Disease super-region and age group in 2016 DALY=disability-adjusted life-year.

About 84·7% of long-term DALYs are due to infectious diseases as a consequence of undernutrition— 87·4% in children aged 28–364 days and 79·7% in children aged 1–4 years—and PEM due to underweight and wasting accounts for the remaining 15·3% of long-term DALYs (figure 3). Most of the acute and long-term sequelae DALYs occurred in the 28–364 days age group (figure 3). The percentage increase in this age group, after accounting for the long-term DALYs compared with acute DALYs, was 50·5% (95% UI 39·8–62·0), whereas the percentage increase in total diarrhoea DALYs in the 1–4 years age group was 32·7% (26·1–40·7). The rate of total diarrhoea DALYs per 1000 was nearly six times higher in the under-1 age group (255·2 per 1000 [224·4–290·3]) than in the 1–4 years age group (42·8 per 1000 [36·6–49·8]).

**Figure 4:**
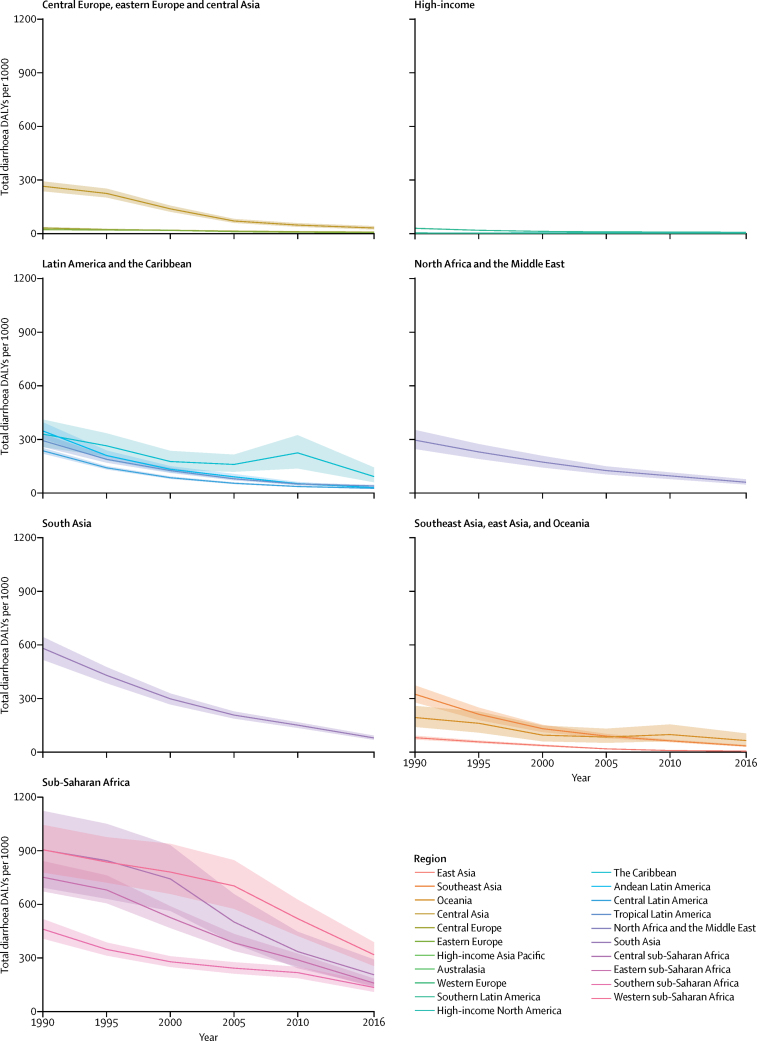
Total diarrhoea DALYs per 1000 globally and by Global Burden of Disease super-region by year among children younger than 5 years DALY=disability-adjusted life-year.

DALYs associated with diarrhoea varied over time (figure 4). At the global level, DALYs attributable to diarrhoea long-term sequelae decreased by 78·7% from 1990 to 2016 (95% UI 69·3–85·5), which was faster than the rate of decline of acute diarrhoea DALYs (70·4% [61·7–76·5]). Total diarrhoea DALYs decreased by more than 90% between 1990 and 2016 in east Asia, Tropical Latin America, and Central Latin America. The greatest reduction in this time period occurred in east Asia (96·5% [95·1–97·5]), Tropical Latin America (90·5% [88·5–93·5]), and Central Latin America (90·2% [88·1–91·9]). The rate of decline was much lower in sub-Saharan Africa (43·8% [26·9–57·2]), with the lowest overall reduction in western sub-Saharan Africa (27·8% [–2·0 to 49·6]), which was not significant. Diarrhoea DALYs increased in high-income North America (23·9% increase [–10·7 to 71·2]) and Australasia (36·6% increase [–10·3 to 105·3]), but these increases were not significant.

## Discussion

By quantifying the long-term sequelae due to growth faltering, our study shows that the global burden of diarrhoea is substantially underestimated when only incidence and mortality are considered. We found that accounting for long-term sequelae increased the number of diarrhoea DALYs among children younger than 5 years by about 40%. After inclusion of these long-term sequelae, diarrhoea moves from the fifth-leading to the third-leading cause of DALYs among children younger than 5 years, surpassing malaria and neonatal encephalopathy in the number of DALYs in this age group.^3^ Most of this long-term burden of diarrhoea is associated with an increased risk of subsequent infectious disease mortality.

We found that wasting, defined as low weight-for-height, is responsible for 40% of the undernutrition-associated diarrhoea DALYs. Among the undernutrition indicators estimated in GBD 2016, the effect of diarrhoea is larger on weight indicators, which could be reflective of short-term deficit, compared with height, which could be a lifelong deficit. This finding is intuitive as diarrhoea is associated with dehydration and immediate weight faltering. The risk of infectious disease mortality due to wasting (low weight-for-height) is much greater than that due to stunting and underweight.^16^ Children that are undernourished or unwell or have been sick recently are at a higher risk of subsequent infection and mortality than are those who are healthy.^18^, ^19^ This fact presents a potentially circular, positive feedback loop in which childhood undernutrition and diarrhoea each increase the risk of the other. When possible, our analysis limited the effect of diarrhoea such that any change in childhood growth indicators occurred after an episode of diarrhoea.

Childhood undernutrition is strongly associated with not only infectious disease mortality but also all-cause child mortality, following a dose-response relationship where the risk of mortality increases as the anthropometric measurements move further from the global mean.^16^ Undernutrition might specifically impair the innate and cell-mediated immune response, cause thymic atrophy, and upset various homoeostatic systems, such as electrolyte balance, glycaemia, and thermoregulation.^20^, ^21^, ^22^ Lower respiratory infections might also be associated with decreased physical growth,^2^ although the mechanism for growth impairment due to respiratory infections might differ from the mechanism for impairment due to diarrhoea, as enteric infections might impede nutrient absorption through inflammation or flattening of intestinal microvilli,^12^, ^23^ pathways that would not be biologically shared in respiratory infections.

We have limited our analysis to the effects of diarrhoea on childhood undernutrition and subsequent morbidity and mortality due to infectious diseases and PEM and have assumed a causal relationship between diarrhoea and childhood growth failure. We chose the outcomes of childhood undernutrition for this study because they are quantifiable and part of GBD 2016. The ability to leverage existing estimates from GBD 2016 is a powerful and unique addition to this analysis. However, evidence exists that undernutrition in childhood might have additional long-term sequelae, such as cognitive impairment, decreased physical fitness in later life, vaccine hyporesponsiveness, Guillain-Barré syndrome, and even chronic diseases in adulthood, such as cardiovascular disease and type 2 diabetes.^11^, ^12^, ^24^, ^25^, ^26^, ^27^ Stunting, low height-for-age, might be a better measure of chronic undernutrition than weight in early childhood, particularly during the first 2 years.^28^ Although stunting might be reversible, it often leads to reduced adult stature unless compensatory growth occurs.^26^, ^28^

Of particular interest is the effect of physical growth stunting on cognitive development. Both cognitive development and physical growth depend on a diverse set of micronutrients and appropriate caloric intake. Some evidence also exists that enteric pathology, with or without diarrhoea, might influence cognitive development outside of the proposed causal pathway between undernutrition and cognitive development, perhaps through chronic immune upregulation and activity or inflammation, where childhood stunting is a comorbidity.^12^, ^29^ Depressed cognitive potential in children might have substantial net effects on economic and social productivity.^30^

We defined diarrhoea as the exposure for impaired physical growth. However, evidence exists that enteric infections leading into environmental enteric dysfunction (EED), even in the absence of diarrhoea, might also be associated with growth faltering.^23^ Enteric infections could more comprehensively define physical growth faltering risk among children younger than 5 years than diarrhoea alone, but quantification of this risk and exposure is difficult. Given the limitations of available data, we believe that diarrhoea episodes provide the best proxy for total enteric infections and accurately reflect the global distribution of the associated burden. Nevertheless, additional research is needed to assess the effect of non-diarrhoea enteric infections, including EED, on child development. Additionally, enteric microbes or families of microbes, such as viruses, bacteria, protozoa, and parasites, with or without diarrhoea, might differentially mediate the relationship between enteric infection and growth impairment. These considerations illustrate that simply accounting for diarrhoea incidence, although the most comprehensive measure available, might be an imprecise metric for the risk of undernutrition due to enteric infections.

Next steps for this work will include quantification of the effect of diarrhoeal diseases and enteric pathogen infections on cognitive development and differentiation of the effect of enteric pathogens on physical growth. We have assumed in this analysis that no difference in the effect of diarrhoea on growth exists between pathogens; however, evidence exists that some pathogens, like *Cryptosporidium*, might have larger effects on growth than might other pathogens.^31^, ^32^ Future iterations will be strengthened with more data than currently available for diarrhoea, diarrhoeal causes, EED, childhood growth, and cognitive development.

The burden of diarrhoea has declined rapidly in many locations in the last 25 years and might meet global targets in under-5 mortality.^6^, ^33^ Treatment, such as oral rehydration solution, has probably played a major role in this progress, but our results suggest that emphasis on diarrhoea and enteric infection prevention, such as provision of safe and reliable water and sanitation and effective vaccines, could have benefits on chronic disease and could lower risk of subsequent infectious disease.^34^, ^35^, ^36^

Our analysis has various potential limitations. The research hypothesis in this analysis is that diarrhoea is causally associated with childhood growth failure and that growth failure is causally associated with infectious disease risk. Assessment of causality in any analysis is difficult and prone to confounding. We did a random-effects meta-analysis, which allows for heterogeneity in the observed effect size that could be due to variation in study populations, exposure, or covariates that were considered in the original analyses.^37^ Still, the results from our analysis show a significant, biologically plausible, temporally structured, and consistent relationship between diarrhoea and childhood growth. Future studies are needed to replicate and strengthen, or refute, this association.

A core component of GBD is to produce comparable estimates of disease burden for every time period, geography, sex, and age group, even in the absence of data to inform these estimates. Our statistical methods are built on predictive validity such that covariates and Bayesian priors help to provide estimates of diarrhoea and undernutrition burden, even in locations without data. All of our modelled estimates include 95% UIs, which reflect our ability to accurately describe disease burden, even in locations without data.

We excluded studies that reported the effect of diarrhoea on weight and height expressed as dichotomous variables. This decision was taken to make an estimate of a continuous change in weight or height, but it reduced the available sample size as many studies reported different measures of association, such as the odds ratio of stunting (HAZ of <2 SDs below the global mean) per diarrhoea episode. Furthermore, we assumed that the distribution of height and weight was normally distributed in every population. This assumption could bias our estimates of the effect of diarrhoea on PEM, depending on whether the distribution of weight, which is left-tailed on the global level, was in fact skewed or not normal in a given population.

Our analysis revealed that, despite decades of progress, the burden of diarrhoea among children younger than 5 years remains sizeable and has been systematically underestimated. Accounting for long-term sequelae of diarrhoea, namely susceptibility to subsequent infectious disease and growth deficiencies, increased estimated diarrhoea DALYs by nearly 40%. These results call for a redoubling of efforts by the international community to prevent diarrhoea and long-term sequelae associated with childhood growth.
