# Supplementary material for: Global disability-adjusted life-year estimates of long-term health burden and undernutrition attributable to diarrhoeal diseases in children younger than 5 years
Source: Lancet Glob Health. 2018 Feb 9;6(3):e255–69. doi: 10.1016/S2214-109X(18)30045-7 (PMC5861379; doi:10.1016/S2214-109X(18)30045-7)
Supplement: Supplementary appendix [file mmc1.pdf]

THE LANCET
Global Health

Supplementary appendix

This appendix formed part of the original submission and has been peer reviewed. We post it as supplied by the authors.

Supplement to: Troeger C, Colombara DV, Rao PC, et al. Global disability-adjusted life-year estimates of long-term health burden and undernutrition attributable to diarrhoeal diseases in children younger than 5 years. *Lancet Glob Health* 2018; **6:** e255–69.

Supplementary material for Global disability-adjusted life-year estimates of long-term health burden and undernutrition attributable to diarrhoeal diseases

This document provides supplementary material for the manuscript *Global disability-adjusted life-year estimates of long-term health burden and undernutrition attributable to diarrhoeal diseases.* The information in the document is intended to provide additional detail for data extraction and modeling strategy.

Contents

Details from Global Burden of Disease 2016 2

Systematic Review and data processing 3

Description of meta-analysis and results 7

Detailed statistical methodology 11

Supplemental results 24

References 33

Details on modeling in GBD 2016

The Global Burden of Disease study 2016 provides comprehensive and internally consistent epidemiological estimates for over 300 causes of death and disability, 90 risk factors, for 195 locations, by year, sex, and four age groups under 5 years old. Detailed descriptions of all GBD methods have been previously published.[1–4] Mortality due to diarrhoeal diseases was estimated using the Cause of Death Ensemble modeling framework (CODEm)[2,5] and diarrhoea incidence, prevalence, and recovery were estimated with DisMod-MR 2.1 (DisMod), a Bayesian meta-regression tool.[6] More detail on diarrhoea modeling in GBD can be found several places including the GBD 2015 diarrhoea capstone manuscript,[1] the GBD 2016 cause of death publication[2] methods appendix (p. 58) and the GBD 2016 non-fatal publication[3] methods appendix (p. 54). The diarrhoea incidence, mortality, years of life lost (YLLs), years lived with disability (YLDs), and disability-adjusted life years (DALYs) estimated in the GBD 2016 framework are referred to herein as *acute DALYs* to differentiate them from DALYs associated with growth impairment which will be referred to as *long-term sequelae DALYs.* DALYs associated with growth impairment will be described in more detail below; we estimated these DALYs as part of the GBD 2016 and estimated these DALYs attributable to diarrhoea in children under-5.

We previously estimated DALYs due to childhood undernutrition in GBD 2016.[4] Childhood undernutrition is modeled using three indicators: stunting, wasting, and underweight. These indicators are based on categorical definitions from the WHO 2006 growth standards for children under-5 using z-scores from an international reference population. For each country, year, sex, and age, we modeled the mean z-score and the prevalence of moderate (less than 2 standard deviations below the reference mean) and severe (less than 3 standard deviations below the reference mean) undernutrition using spatiotemporal Gaussian process regression (ST-GPR). More detail on modeling childhood growth failure in GBD 2016 can be found on p. 73 of the risk factors publication methods appendix.[4]

Childhood undernutrition is associated with three outcomes in GBD 2016: diarrhea, lower respiratory infections, and measles (risk-outcome pairs). The relative risks for these risk-outcome pairs were derived from a pooled cohort analysis.[4,7] Undernutrition indicators are highly correlated in a population and so the relative risks were adjusted to account for covariance between them by simulating a joint distribution of the three indicators using the distributions extracted from the countries that were included in the original relative risk cohort analysis (individual-level data from Demographic and Health Survey [DHS] microdata). We extracted the interaction terms from the analysis and adjusted the RRs by minimizing the error between the crude (from pooled cohort) and expected RRs derived from our simulation (more information available in the GBD 2015 risk factors manuscript, methods appendix p. 34).[8]

Details on modeling mortality, morbidity, YLLs, YLDs, and DALYs due to measles and lower respiratory infections can be found in the GBD 2016 cause of death methods appendix (p.83 and p. 67, respectively)[2] and in the GBD 2016 non-fatal methods appendix (p.97 and p. 66, respectively),[3] and lower respiratory infections in the GBD 2015 LRI publication.[9]

We attributed 100% of protein-energy malnutrition (PEM) to childhood wasting and underweight but not stunting. PEM mortality was modeled as a sub-category of total nutritional deficiencies in CODEm and PEM prevalence is back-calculated as a composite of marasmus and kwashiorkor using the case fatality ratio and mean duration of illness (GBD 2016 cause of death manuscript methods appendix p. 117).[2]

Systematic Review

Our primary long-term outcomes of interest were changes in Z-scores of height or length for age (HAZ), weight for age (WAZ), and weight for height (WHZ) subsequent to DD. We defined mild, moderate, and severe stunting, underweight, and wasting as -1 to -2, -2 to -3, and <-3 Z-scores of HAZ,

WAZ, and WHZ, respectively. When possible, HAZ, WAZ, and WHZ were defined according to the World Health Organization (WHO) 2006 growth charts[10], rather than the CDC 2000[11] or the 1977 National Center for Health Statistics (NCHS) growth charts. We selected these outcomes because they are health related, quantifiable, have sufficient literature or available microdata for meta-analyses, and are available in the Global Burden of Disease Study (GBD).[12–14]

Search strategy

We conducted a series of systematic reviews to address three questions to inform our DD DALYs estimates. We sought to quantify the change in HAZ, WAZ, and WHZ due per day of early childhood (under 5 years of age) diarrhoea. Based on these questions, we performed detailed literature searches using EBSCO, Embase, and PubMed databases (Supplementary Table 1). We also examined the references list of a 2015 meta-analysis for additional publications[15] and performed analyses on microdata that we obtained for this study. There was no restriction on publication date. The search terms used were the following.

PubMed:
(diarrh[Title/Abstract] or "gastroenteritis"[Title/Abstract] or "acute enteritis"[Title/Abstract] or gastrit*[Title/Abstract]) and ("intellectual impairment"[Title/Abstract] or "cognitive impairment"[Title/Abstract] or "child development"[Title/Abstract] or "personality development"[Title/Abstract] or "academic achievement"[Title/Abstract] or "verbal fluency"[Title/Abstract] or "learning"[Title/Abstract] or memory[Title/Abstract] or "psychosocial"[Title/Abstract] or "developmental disorder"[Title/Abstract])*

Embase:
(diarrh:ab or diarrhoea:ab or 'gastroenteritis':ab or 'acute enteritis':ab or gastrit*:ab) and (stunting:ab or wasting:ab or malnutrition:ab or 'child growth':ab or 'child nutrition':ab or underweight:ab or weight:ab) and ('prospective study':ab or 'retrospective study':ab or 'cohort analysis':ab or 'case control study':ab or 'randomized control trial':ab) and [humans]/lim*

EBSCO:
AB (diarrhoea or diarrhoeal or diarrhoea or gastroenteritis) AND AB (stunting or wasting or development or growth or underweight or malnutrition or "height" or "weight") AND AB (child or infant or baby)

Data extraction

From our search terms, we identified 1355 titles from PubMed, 390 titles from Embase, and 1011 titles from EBSCO. We screened titles and abstracts identified in our systematic review to identify articles for a full-text review. After deduplication, we screened 539 articles and identified articles that provided information on weight, height, and diarrhoea. After full text review, we extracted information from 17 studies that provided height or weight change due to diarrhoea (**SI Table 1**). To extract information from the articles, we used a standardized database to extract the title, journal, volume, issue, pages, publication date, abstract, location(s), study year(s), exposure and outcome ages, descriptions of exposure and outcome measures, cohort identifiers, table or figure numbers, point estimates, and standard errors (SE) or confidence intervals (CI). When crude and adjusted estimates were provided, we exacted both. We did not assess study quality.

Exclusion criteria

We excluded exposures, whether DD or undernutrition, that occurred older than age five and outcomes assessed older than age 12. We limited our analysis to studies which could assess nutritional status in terms of continuous HAZ, WAZ, and WHZ. We excluded studies that limited these assessments to dichotomized variables such as "stunting", "underweight", and "wasting." We also excluded studies that solely assessed micronutrient supplementation, community health education, psycho social stimulation, WASH and nutritional interventions, helminthic infection, and breastfeeding as DD risk factors. We had no exclusions for publication date or language.

Additional data sources

We supplemented the literature review above with original analyses of individual-level observational data from cohort studies (**SI Table 1**). Changes in HAZ, WAZ, and WHZ per day of diarrhoea were assessed in a birth cohort in Vellore, India, a *Cryptosporidium* study in Bangladesh to assess the association between diarrhoea and undernutrition, The Performance of Rotavirus and Oral Polio Vaccines in Developing Countries (PROVIDE) Study in Bangladesh, and a birth cohort in Bangladesh.[16–19] In addition, we included individual level data from the Etiology, Risk Factors, and Interactions of Enteric Infections and Malnutrition and the Consequences for Child Health (MAL-ED) Study child cohort.[20]

For each dataset, we calculated the difference in HAZ, WAZ, and WHZ between consecutive anthropometric measures. We used panel regression to estimate the average change in these measures per day of diarrhoea *between* anthropometric measurements, adjusted for the number of days between measures, the baseline HAZ/WAZ/WHZ measure, and the child's age in months. All of our panel regressions used a maximum likelihood random-effects estimator.

SI Table 1. Source data list.

First Author	Location	Years	Age range (years)	Input	Type
Assis[21]	Brazil	1990 - 1991	0.5 to 4	HAZ, WAZ	Literature
Bairagi[22]	Bangladesh	1975 - 1976	1 to 4	HAZ, WAZ	Literature
Becker[23]	Bangladesh	1978 - 1979	0.5 to 2.7	WAZ	Literature
Black[24]	Bangladesh	1978 - 1979	0.5 to 4	HAZ, WAZ	Literature
Donowitz[16]	Bangladesh	2008 - 2014	0 to 6	HAZ, WAZ, WHZ	Microdata
Faruque[17]	Bangladesh	2014 - 2016	0 to 2	HAZ, WAZ, WHZ	Microdata
Kattula[18]	India	2009 - 2013	0 to 3	HAZ, WAZ, WHZ	Microdata
Kirkpatrick[19]	Bangladesh	2011 - 2014	0 to 2	HAZ, WAZ, WHZ	Microdata
Kotloff[25]	The Gambia, Mali, Kenya, Mozambique, Pakistan, India, Bangladesh	2007 - 2011	0 to 5	HAZ	Literature
Lee[26]	Peru	2002 - 2006	0 to 6	HAZ, WAZ	Literature
Lima[27]	Brazil	1989 - 1993	0 to 3	HAZ, WAZ	Literature
MAL-ED Network[28]	Bangladesh, Brazil, India, Nepal, Pakistan, Peru, South Africa, Tanzania	2009-2014	0 to 2	HAZ, WAZ, WHZ	Microdata
Moffat[29]	Nepal	1995	0 to 5	HAZ, WAZ	Literature
Molbak[30]	Guinea-Bissau	1987 - 1990	0 to 2	HAZ	Literature
Mondal[31]	Bangladesh	1999 - 2002	2 to 5	HAZ, WAZ	Literature
Moore[32]	Brazil	1989 - 1998	0 to 2	HAZ	Literature
Moore[33]	Brazil	1989 - 2000	0 to 10	HAZ, WAZ, WHZ	Literature
Moy[34]	Zimbabwe	1992 - 1993	0 to 1.2	HAZ, WAZ	Literature
Sawadogo[35]	Burkina Faso	2003 - 2005	0.5 to 2	HAZ	Literature
Walker[36]	Jamaica	1989 - 1991	0.8 to 2	HAZ, WAZ	Literature
Weisz[37]	Malawi	2009 - 2010	0.5 to 1.5	HAZ, WAZ	Literature
Wierzba[38]	Egypt	1993 - 1995	1 to 3	HAZ, WAZ, WHZ	Literature

Data transformations

For the diarrhoea to undernutrition analysis, we aimed to identify data that measured the change in nutritional status (e.g., HAZ) per day of diarrhoea. For estimates of change in nutritional status per episode of diarrhoea, the coefficient and standard error (SE) were divided by the mean number of days per episode of DD in that study. If the authors provided a range, we used the median of that range. When only a lower limit was provided, we used that lower limit. We assumed an average duration of 4.3 days per diarrhoeal episode when the days per episode could not be estimated from the study, consistent with the mean duration of diarrhoea episodes in GBD 2016.[3] When estimates were based on diarrhoea prevalence (percent of days with diarrhoea), we multiplied the estimate by the number of days in that period, while assuming 30 days per month. Estimates based directly on changes in anthropometric measures (i.e., height in cm) were adjusted by dividing the coefficients and SE by the

standard deviation (SD) for that metric at a particular age from the WHO Growth Charts.[10] The age was defined as the midpoint of the age range in the study and female growth charts were used for mixed populations of males and females.

We needed beta coefficients and SEs for our meta-analyses. When possible, we converted correlation coefficients to beta coefficients:

$$\beta = Correlation(X, Y) * \frac{SD(X) * SD(Y)}{Variance(X)}$$

When a publication provided a beta coefficient and p-value, we calculated the standard error as:

$$Standard\ Error = \frac{\beta}{Z}$$

Where:[39]

$$Z = -0.862 + (0.743 - 2.404 * \ln(p))^{0.5}$$

Meta-analysis

We performed a series of random-effect meta-analyses to determine the effect per day of diarrhoea on height-for-age, weight-for-age, and weight-for-height among children under-5 (**SI Figures 1-3**). We used the most detailed available information from the extracted literature sources in terms of age and location in the meta-analyses. All meta-analyses were performed in R v3.3.2 using the 'metaphor' package.

SI Figure 1. Forest plot of the relationship between height-for-age z-scores (HAZ) per day of diarrhoea. The random-effects meta-analysis uses input data at the finest granularity reported by study authors. Where individual-level data were available, a summary value by age group was used.

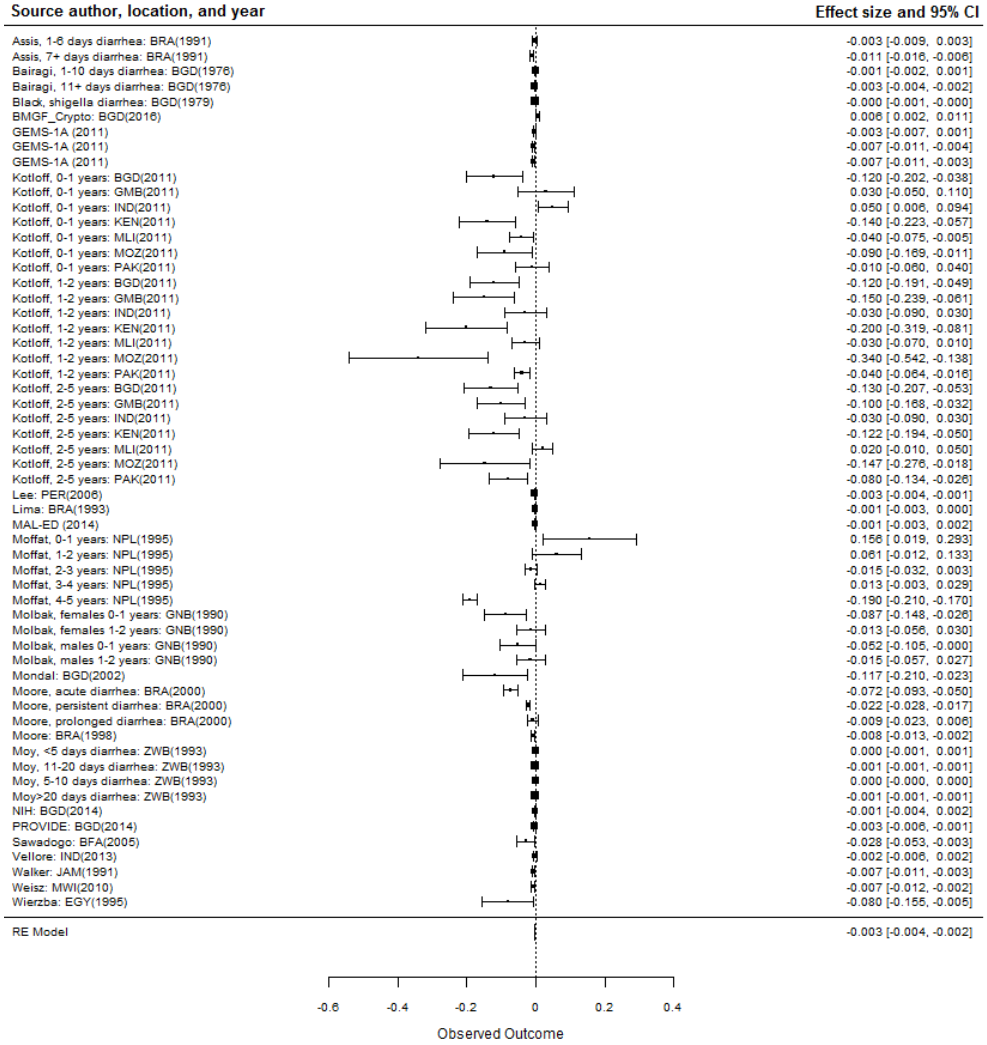

SI Figure 2. Forest plot of the relationship between weight-for-age z-scores (WAZ) per day of diarrhoea. The random-effects meta-analysis uses input data at the finest granularity reported by study authors. Where individual-level data were available, a summary value by age group was used.

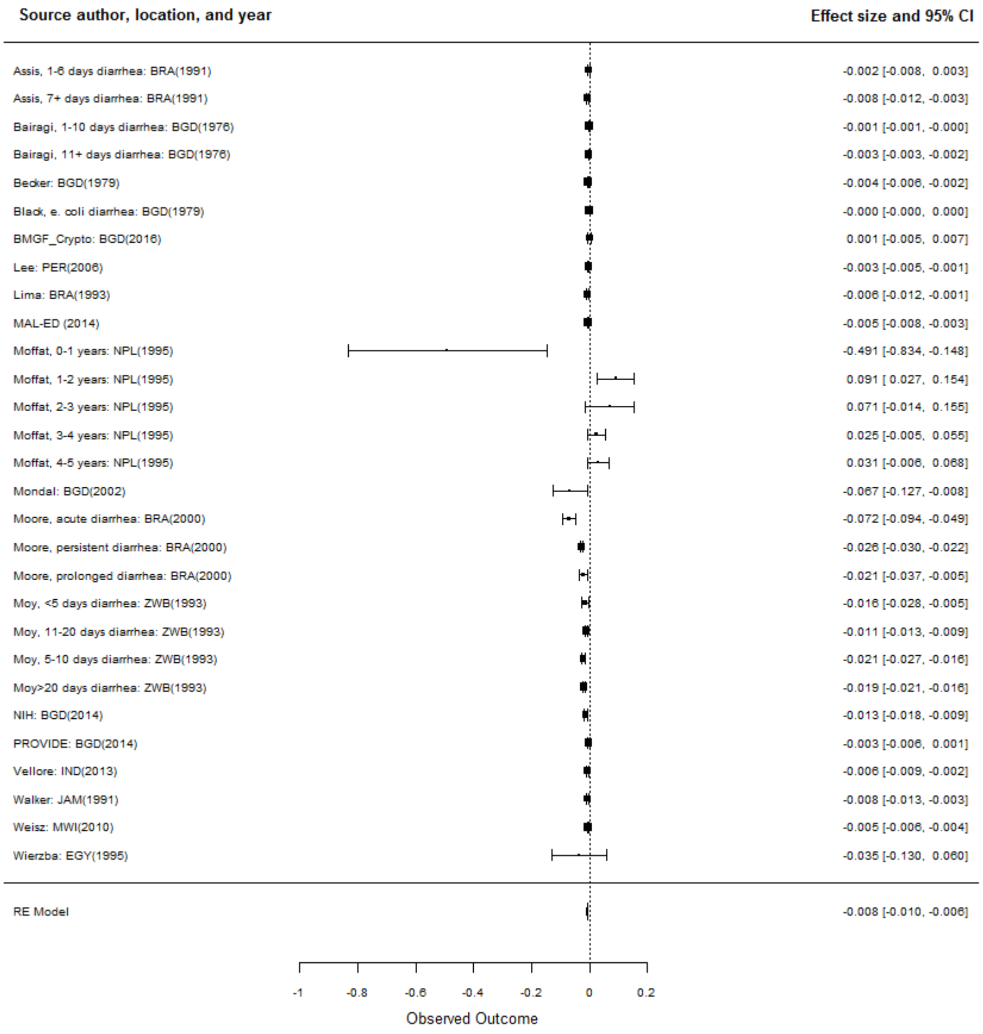

SI Figure 3. Forest plot of the relationship between weight-for-height z-scores (WHZ) per day of diarrhoea. The random-effects meta-analysis uses input data at the finest granularity reported by study authors. Where individual-level data were available, a summary value by age group was used.

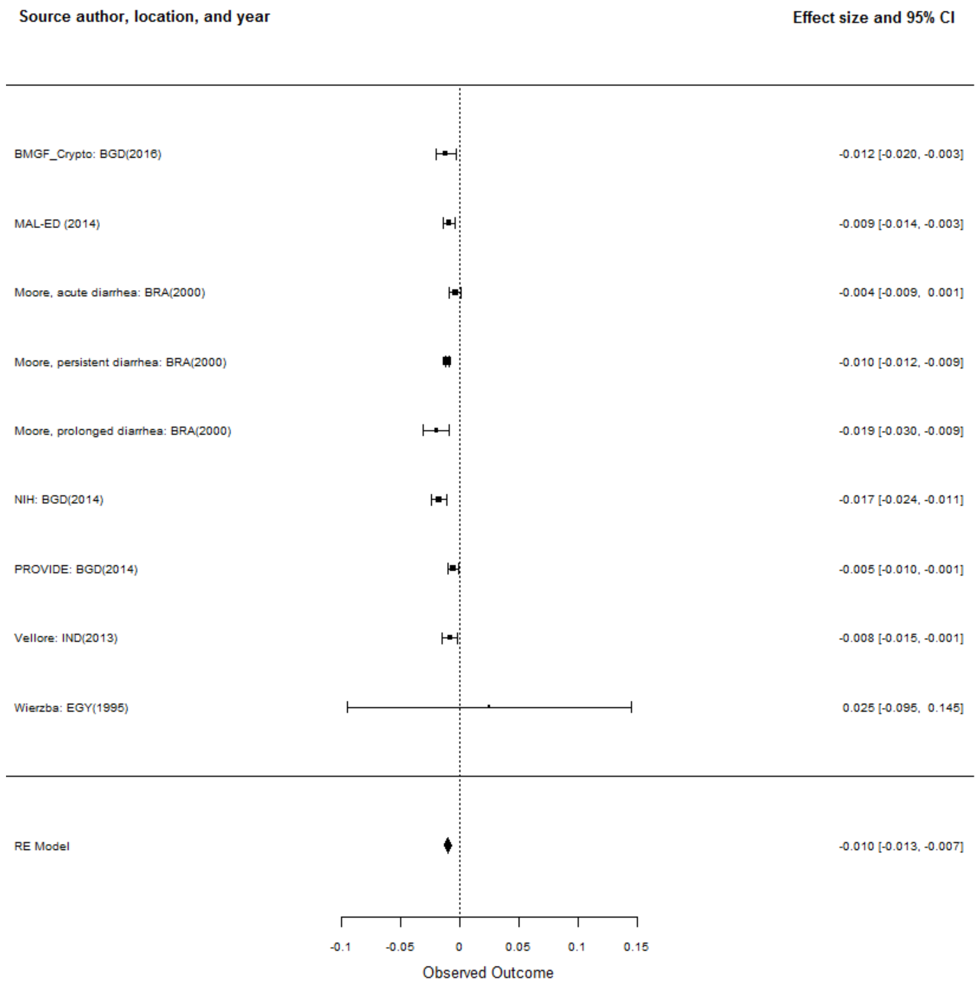

Statistical analysis

Flowchart 1. Analytic flowchart for estimating the effect of diarrhoea on physical growth and childhood undernutrition and subsequent disability-adjusted life years (DALYs) associated with undernutrition due to diarrhoeal diseases.

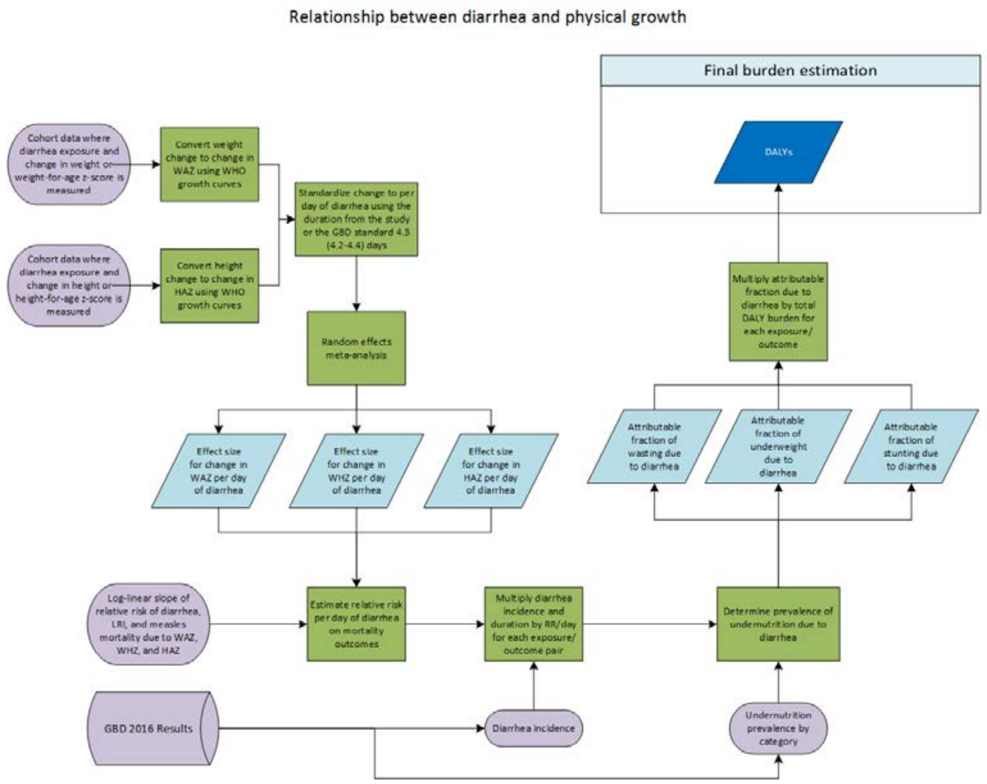

Using the results of our systematic review and microdata analyses, we conducted a random effects meta-analysis. When available, we used multivariate-adjusted estimates rather than crude estimates from the literature review in our analyses.

Diarrhoea-associated undernutrition. For the DD to undernutrition analysis, we estimated the log-linear relative risk (RR) of disease and mortality for diarrhoea, lower respiratory tract infection (LRI), and measles per unit change in HAZ, WAZ, and WHZ based on hazard ratios reported in a 2013 pooled analysis **(SI Figure 5)**.[40] Although hazard ratios may not be equivalent to a risk ratio in many circumstances, we assume that the hazard ratios can be treated similarly to risk ratios in assessing the

association between a risk and an outcome in estimating an attributable burden.[41] Hazard ratios will be referred to generally as a relative risk (RR) in this manuscript. The relative risks for stunting and wasting are adjusted to account for their covariance with each other and with underweight.[14] Undernutrition indicators are highly correlated in a population and so the relative risks were adjusted to account for covariance between them by simulating a joint distribution of the three indicators using the distributions extracted from the countries that were included in the original relative risk cohort analysis (individual-level data from Demographic and Health Survey [DHS] microdata). We extracted the interaction terms from the analysis and adjusted the RRs by minimizing the error between the crude (from pooled cohort) and expected RRs derived from our simulation (more information available in the GBD 2015 risk factors manuscript, methods appendix p. 34).[8] We then calculated an outcome-specific population attributable fraction (PAF) for each age group under 5 years and for each year, sex, and location where:

$$PAF = 1 - \frac{1}{Diarrhoea\ incidence\ *\ Episode\ Duration\ *\ \frac{\Delta Zscore}{day\ of\ diarrhoea} * \mathrm{RR}}$$

Where *Diarrhoea incidence* is the modeled number of episodes per child-year in GBD 2016, *Episode duration* is the mean duration in days per diarrhea episode, Δ*Zscore* is the unit change per day of diarrhea, and *RR* is the relative risk of each outcome per unit change in z-score. The overall undernutrition PAF for each outcome was calculated as:

$$PAF_{malnutrition} = 1 - [(1 - PAF_{WAZ}) * (1 - PAF_{HAZ}) * (1 - PAF_{WHZ})]$$

We multiplied the DD, LRI, and measles undernutrition PAFs by the GBD 2016 total estimated DALYs associated with those outcomes.[42] Uncertainty in the final DALYs estimates was calculated using 1000 draws of the input parameters including DD incidence, effect size from the meta-analyses, and relative risk of the outcome. Our final DALYs point estimate is the mean DALYs estimate, with the upper and lower uncertainty intervals (UI) defined by the 2.5 and 97.5 percentiles of the estimates, respectively.

To estimate the attribution of diarrhoea on protein-energy malnutrition, we used a counterfactual approach to define the percent change in the cumulative density from a normal distribution. From GBD 2016, we used the estimated prevalence of mild, moderate, and severe underweight and wasting for each age group under five, each location, both sexes, and the years 1990, 1995, 2000, 2005, 2010, and 2016. We converted the prevalence of undernutrition categories to z-scores. We then estimated the z-score shift per day of diarrhoea based on the GBD 2016 estimated diarrhoea incidence and the effect sizes from our meta-analysis. We defined the counterfactual prevalence of wasting or stunting as the difference in the observed and z-score-shifted prevalences. The PAF was the percent difference in the observed and counterfactual prevalence estimates (**SI Figure 4**).

We estimated protein energy undernutrition (PEM) DALYs attributable to DD using 1000 draws of DD incidence, PEM DALYs, and the prevalence of underweight and wasted children from GBD 2016. We defined underweight and wasting as the cumulative prevalence of mild, moderate, and severe measures. We multiplied the estimated change in WAZ and WHZ due to one day of diarrhoea from the meta-analysis by the estimated diarrhoea incidence from GBD 2016 times the duration in days per episode (4.3 days on average, range: 4.2-4.4 days)[43] to calculate the change in underweight and wasting due to diarrhoea, respectively. We defined the counterfactual prevalence of underweight in children as:

$$Prevalence_{Counterfactual} = Prevalence\ + \frac{\Delta Zscore}{day\ of\ diarrhea} * Diarrhea\ Incidence * Episode\ Duration$$

and calculated the PAF as

$$PAF = \frac{Prevalence(Counterfactual) - Prevalence\ (GBD\ 2016\ Estimate)}{Prevalence\ (GBD\ 2016\ Estimate)}$$

The PAF for wasting was calculated in a parallel manner using WHZ. PEM DALYs attributable to DD were calculated by multiplying the PAF by the total PEM DALYs, again taking the mean as our point estimate and the 2.5 and 97.5 percentiles as the lower and upper UI limits. All analyses used two-sided statistical tests and were conducted using Stata/SE 13.1 and R version 3.3.2.

SI Figure 4. This figure shows the analytic strategy for evaluating the counter-factual attributable fraction for the impact of diarrhoea on weight-for-age and weight-for-height. The cumulative density curves show the observed (blue) and counterfactual prevalence. The counterfactual curve is set to mean 0 and standard deviation 1 and the observed curve is a hypothetical population with a mean of -1 and standard deviation of 1. The prevalence of wasting in this example is the area below -2 standard deviations. Shifting the curve to the right (counterfactual curve) decreases the prevalence of wasting. The percent difference in the prevalence of wasting (dashed lines) is the population attributable fraction.

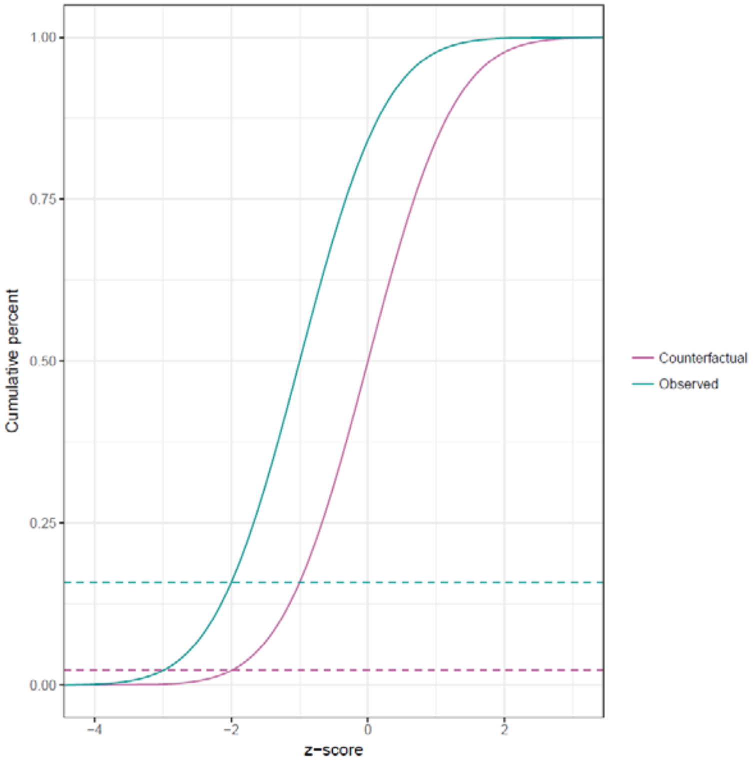

SI Figure 5. The adjusted hazard ratios for mortality, from Olofin et al 2013, for each risk-outcome pair is shown. Fit was evaluated in log-space (primary y-axis) and the plots also show the exponentiated prediction (secondary y-axis). The log-linear slope represents the per z-score unit change in the hazard ratio of mortality.

Comment [CT1]: Cite this!

A)

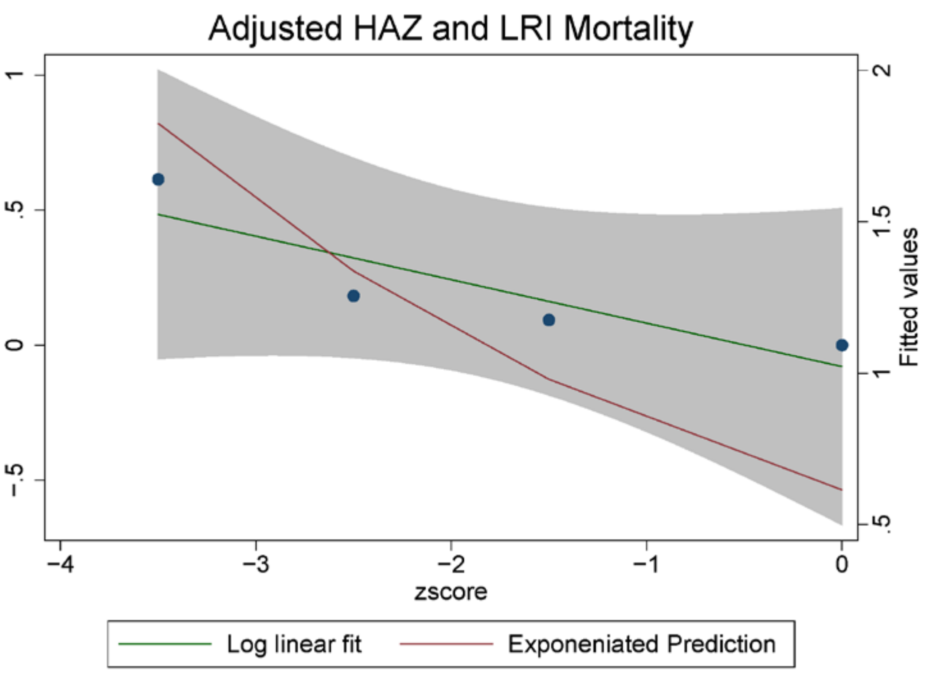

B)

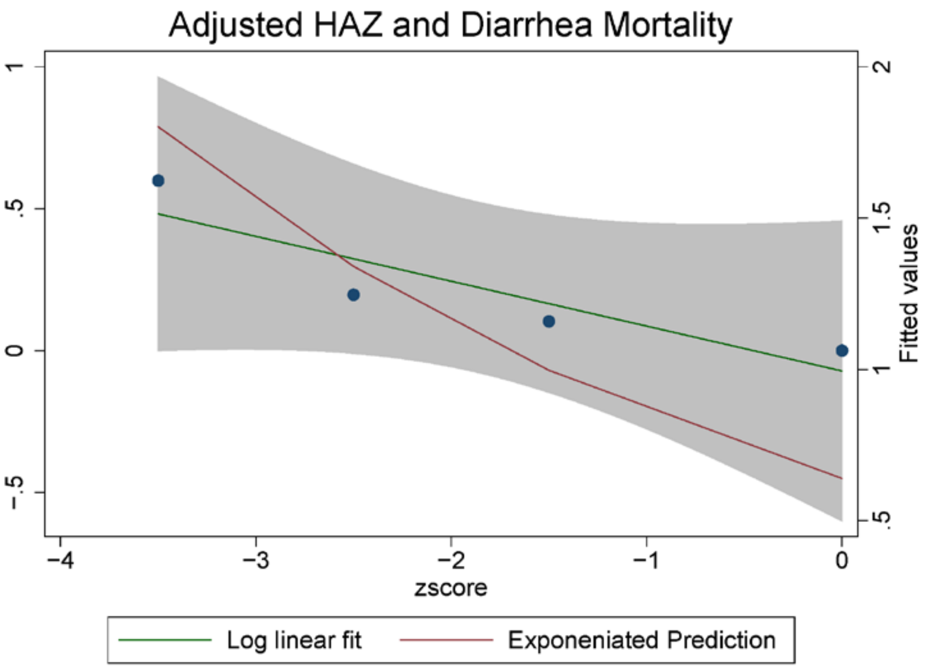
Adjusted HAZ and Diarrhea Mortality
zscore
Fitted values
Log linear fit
Exponeniated Prediction

c)

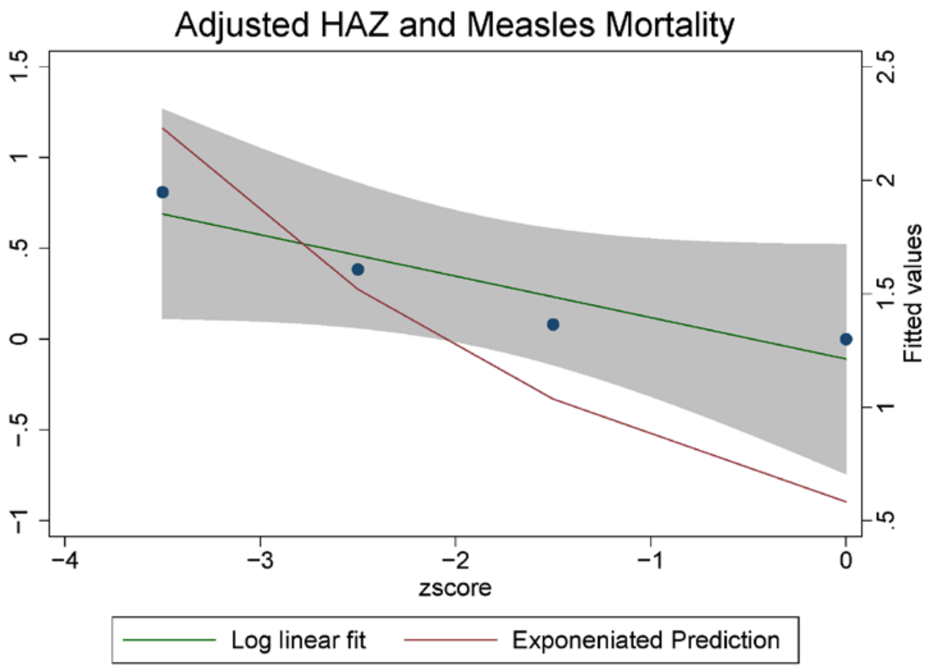
Adjusted HAZ and Measles Mortality
−1
−.5
0
.5
1
1.5
.5
1
1.5
2
2.5
Fitted values
−4
−3
−2
−1
0
zscore
Log linear fit
Exponeniated Prediction

D)

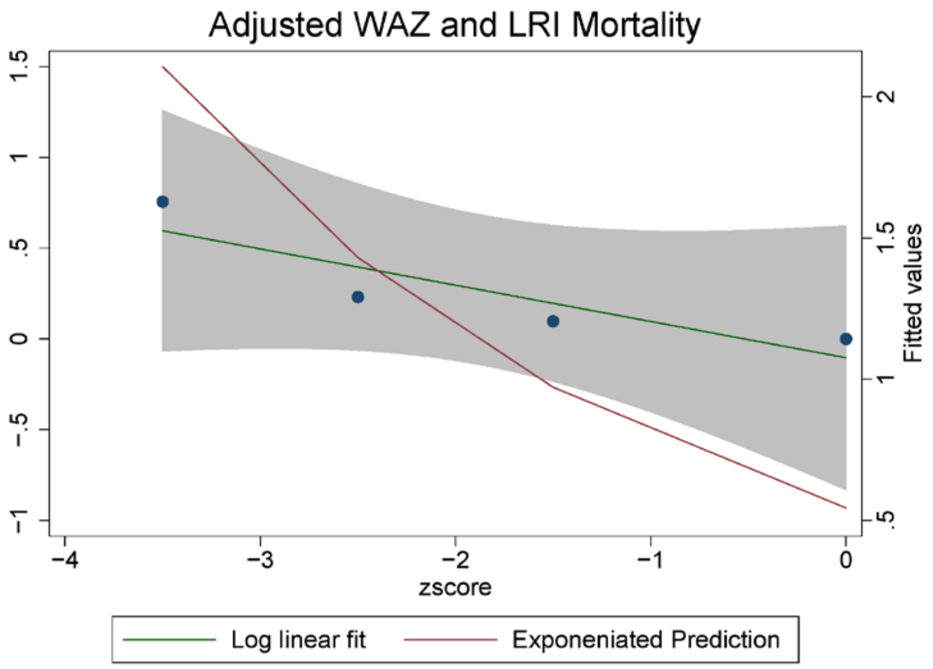

Adjusted WAZ and LRI Mortality
1.5
1
.5
0
−.5
−1
2
1.5
1
.5
Fitted values
−4
−3
−2
−1
0
zscore
Log linear fit
Exponeniated Prediction

385 **E)**

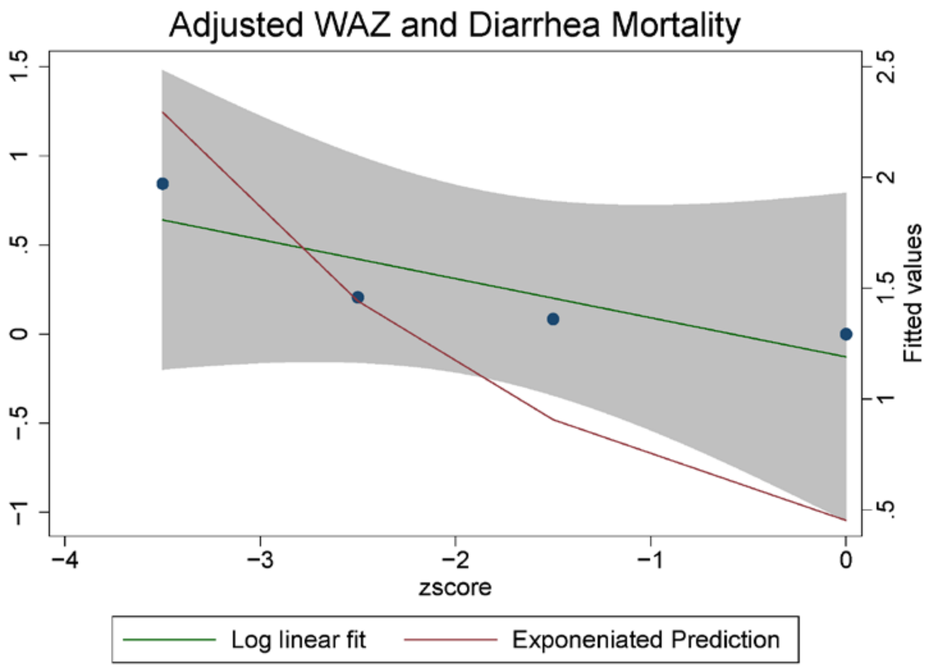
Adjusted WAZ and Diarrhea Mortality
1.5
1
.5
0
−.5
−1
2.5
2
1.5
1
.5
Fitted values
−4
−3
−2
−1
0
zscore
Log linear fit
Exponeniated Prediction

F)

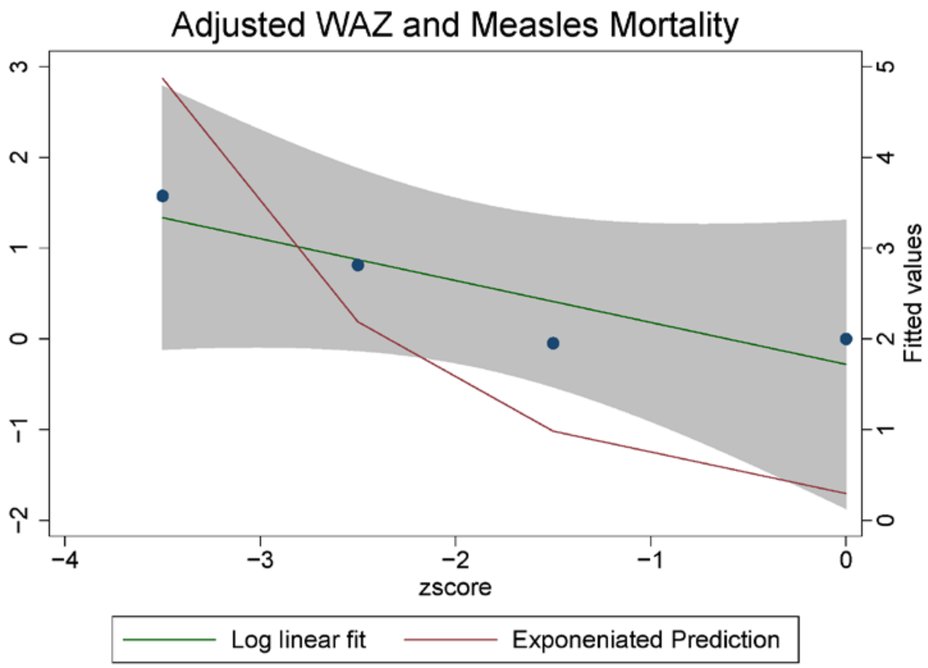

Adjusted WAZ and Measles Mortality
3
2
1
0
−1
−2
5
4
3
2
1
0
Fitted values
−4
−3
−2
−1
0
zscore
Log linear fit
Exponeniated Prediction

G)

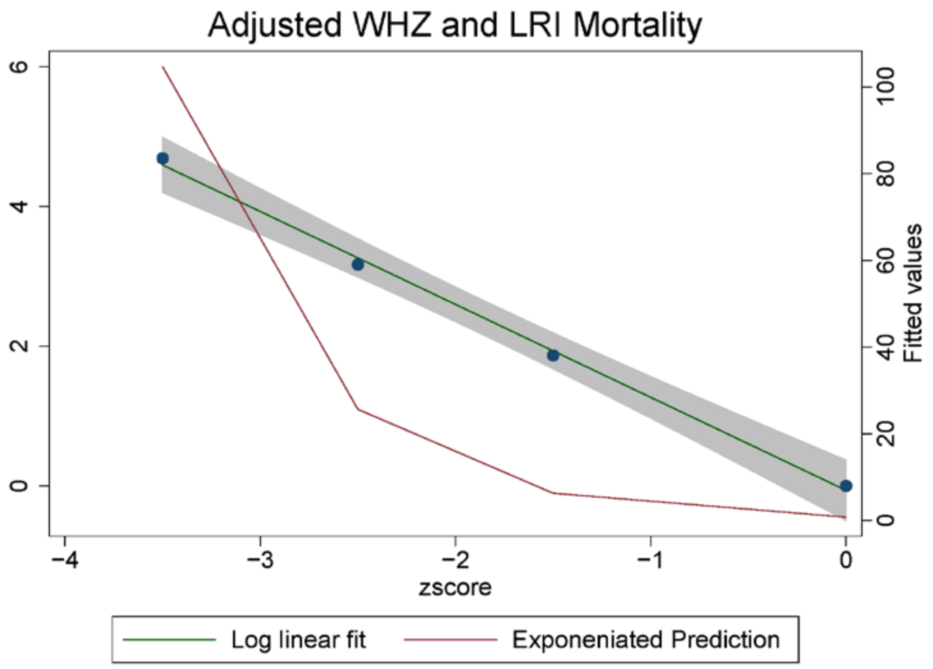
Adjusted WHZ and LRI Mortality
6
4
2
0
100
80
60
40
20
0
Fitted values
−4
−3
−2
−1
0
zscore
Log linear fit
Exponeniated Prediction

390

H)I)I)

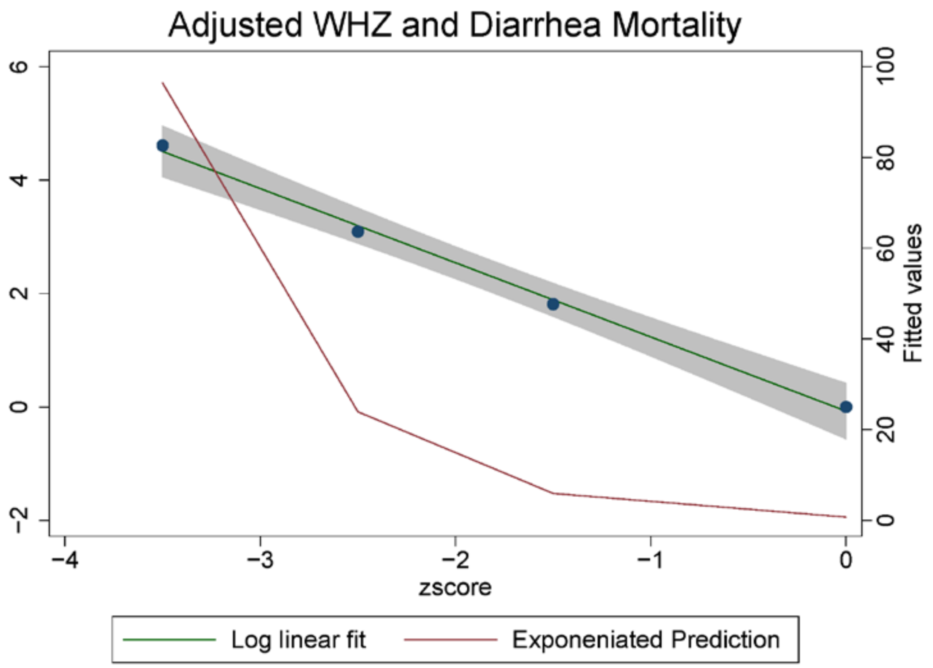

Adjusted WHZ and Diarrhea Mortality
6
4
2
0
−2
100
80
60
40
20
0
Fitted values
−4
−3
−2
−1
0
zscore
Log linear fit
Exponeniated Prediction

I)

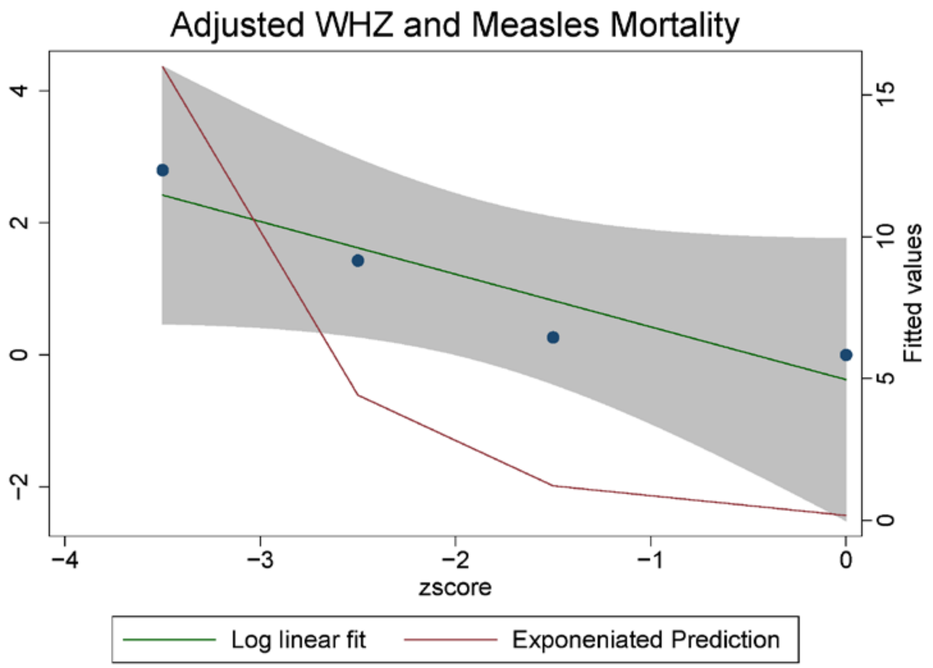
Adjusted WHZ and Measles Mortality
4
2
0
−2
15
10
5
0
Fitted values
−4
−3
−2
−1
0
zscore
Log linear fit
Exponeniated Prediction

Supplementary results

Table 1. Effect sizes from the systematic review and meta-analyses of the effect of diarrhoea on physical growth.

Measured Association	Estimate	Standard Error	Lower	Upper	p-value
Diarrhoea and HAZ	-0.00327	0.000433	-0.00412	-0.00242	4.43E-14
Diarrhoea and WAZ	-0.00774	0.000982	-0.00966	-0.00581	3.19E-15
Diarrhoea and WHZ	-0.00964	0.001482	-0.01254	-0.00673	7.78E-11

Acute diarrhoeal disease DALYs per 1000 under-5 in 2016. This map shows the acute diarrhoea DALYs (diarrhoea incidence and mortality) per 1,000 children under-5 years old in 2016.

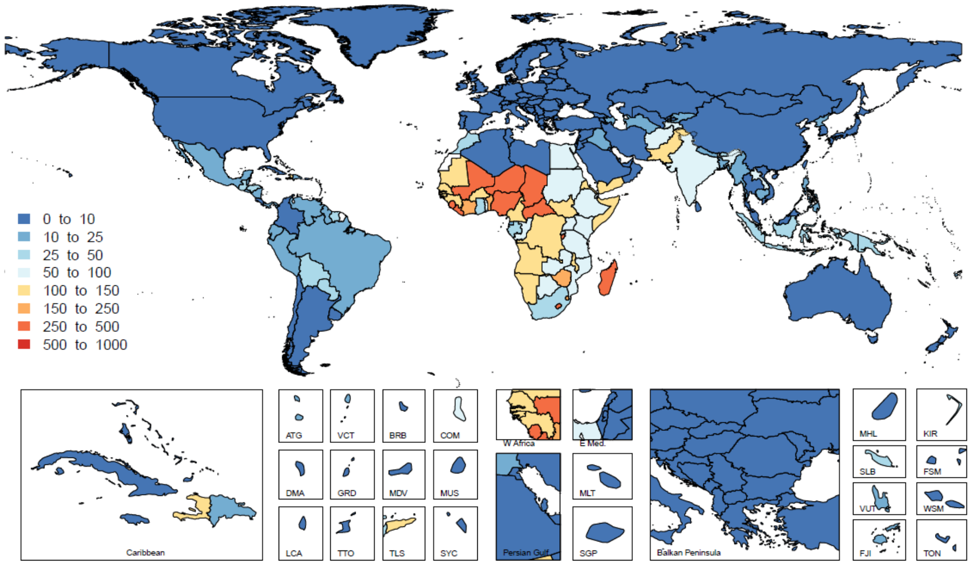

Long-term sequelae diarrhoeal disease DALYs per 1000 under-5 in 2016. This map shows long-term sequelae diarrhoea DALYs, due to growth impairment, per 1,000 children under-5 years old in 2016.

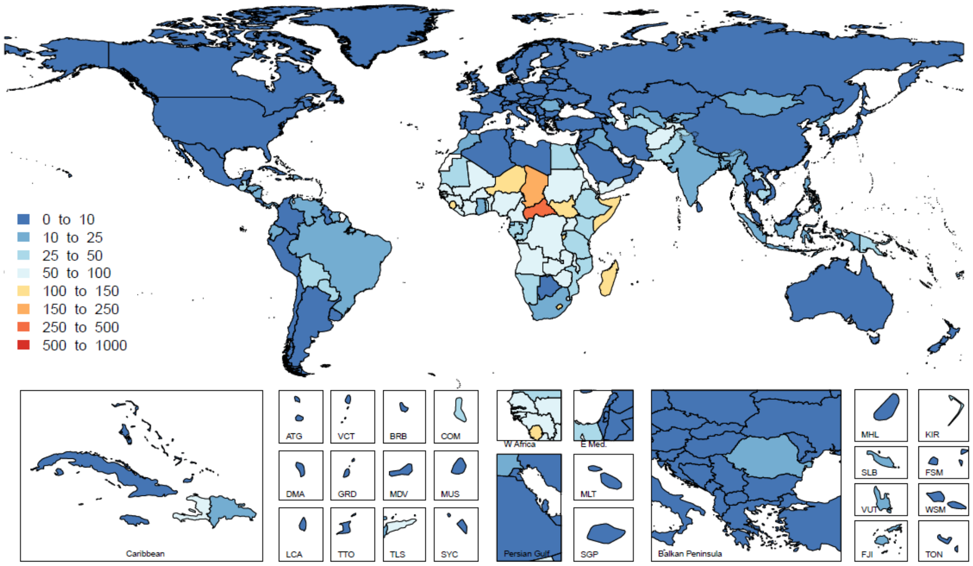

Diarrhoea DALYs per 1000 by sequelae source and GBD super-region among children under 5 years old in 2016. Acute DALYs represent the burden of diarrhoea before considering the long-term sequelae associated with physical growth impairment. Diarrhoea WHZ, WAZ, and WHZ DALYs represent the burden of diarrhoea due to its effect on weight and height and the subsequent risk of morbidity and mortality due to impaired physical growth. Protein-energy malnutrition (PEM) DALYs represent the burden of disease due to low weight caused by diarrhoea. Countries are ordered from top to bottom by the total number of DALYs due to diarrhoeal diseases in 2016.

A

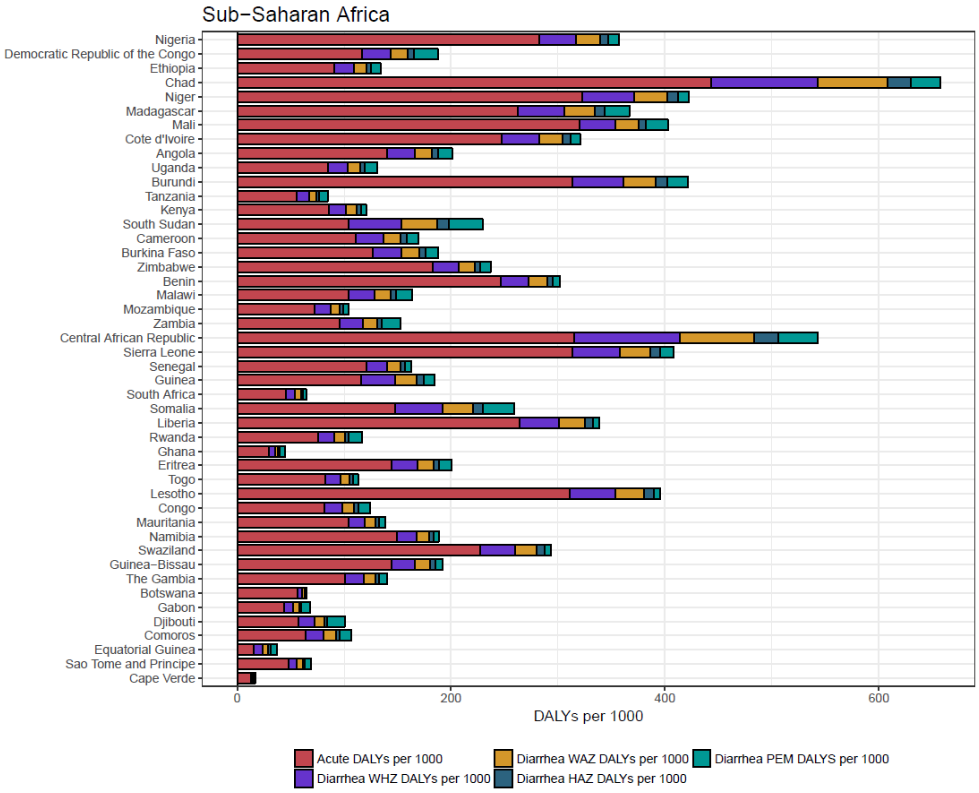

B

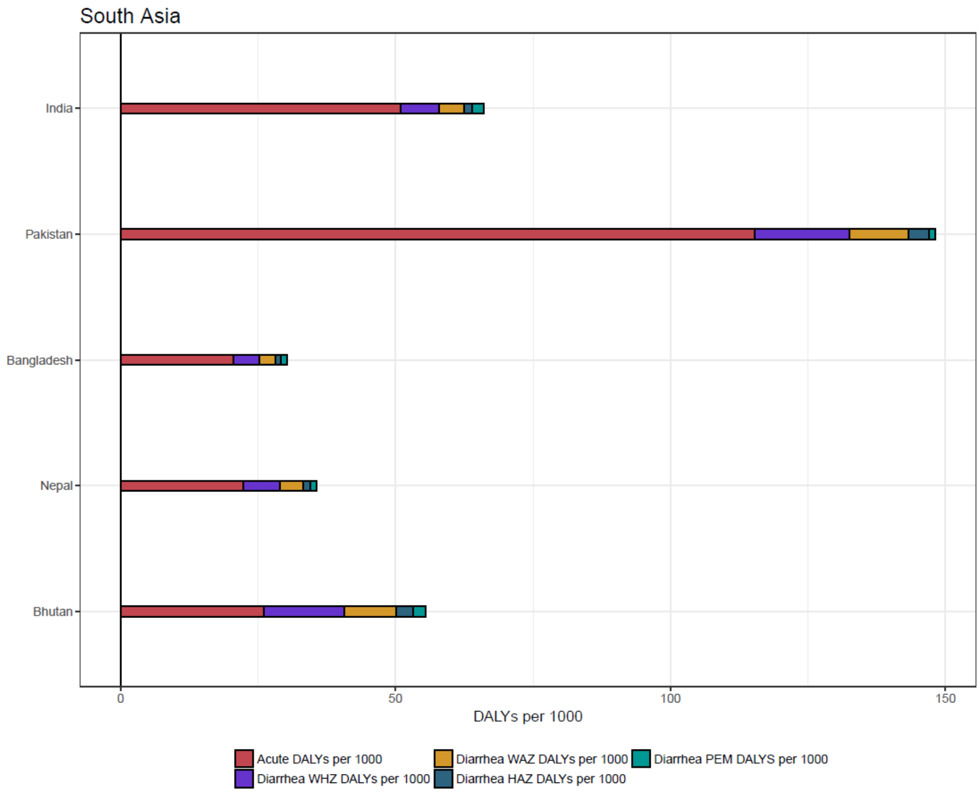

South Asia
India
Pakistan
Bangladesh
Nepal
Bhutan
0
50
100
150
DALYs per 1000
Acute DALYs per 1000
Diarrhea WHZ DALYs per 1000
Diarrhea WAZ DALYs per 1000
Diarrhea HAZ DALYs per 1000
Diarrhea PEM DALYS per 1000

C

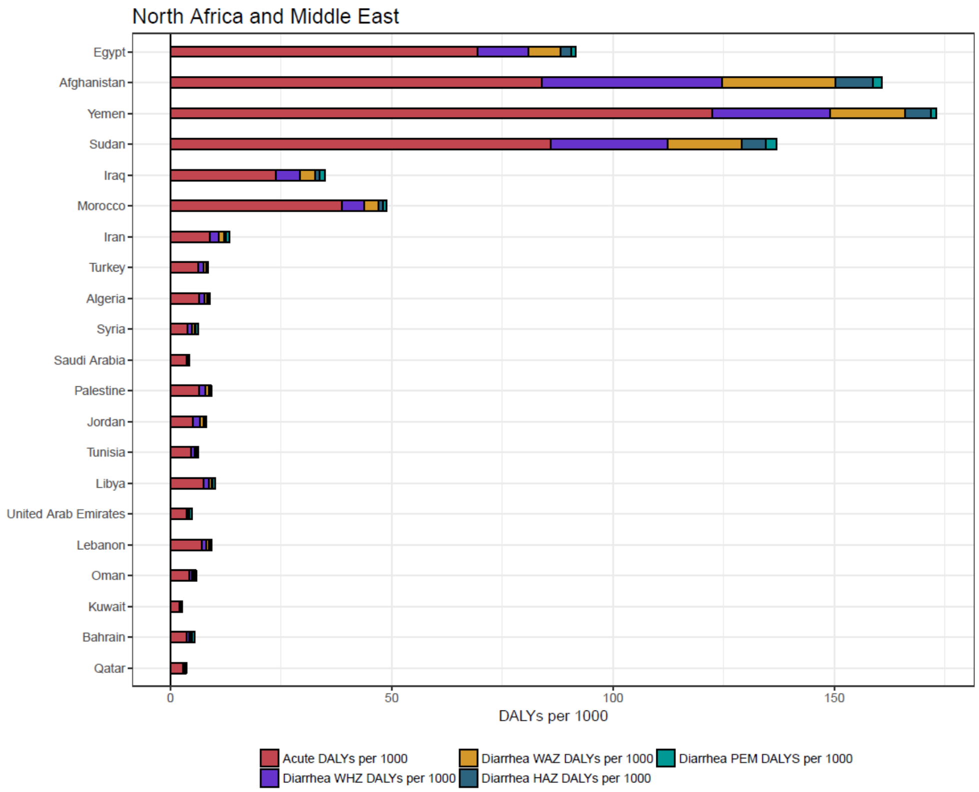
North Africa and Middle East
Egypt
Afghanistan
Yemen
Sudan
Iraq
Morocco
Iran
Turkey
Algeria
Syria
Saudi Arabia
Palestine
Jordan
Tunisia
Libya
United Arab Emirates
Lebanon
Oman
Kuwait
Bahrain
Qatar
0
50
100
150
DALYs per 1000
Acute DALYs per 1000
Diarrhea WHZ DALYs per 1000
Diarrhea WAZ DALYs per 1000
Diarrhea HAZ DALYs per 1000
Diarrhea PEM DALYS per 1000

D

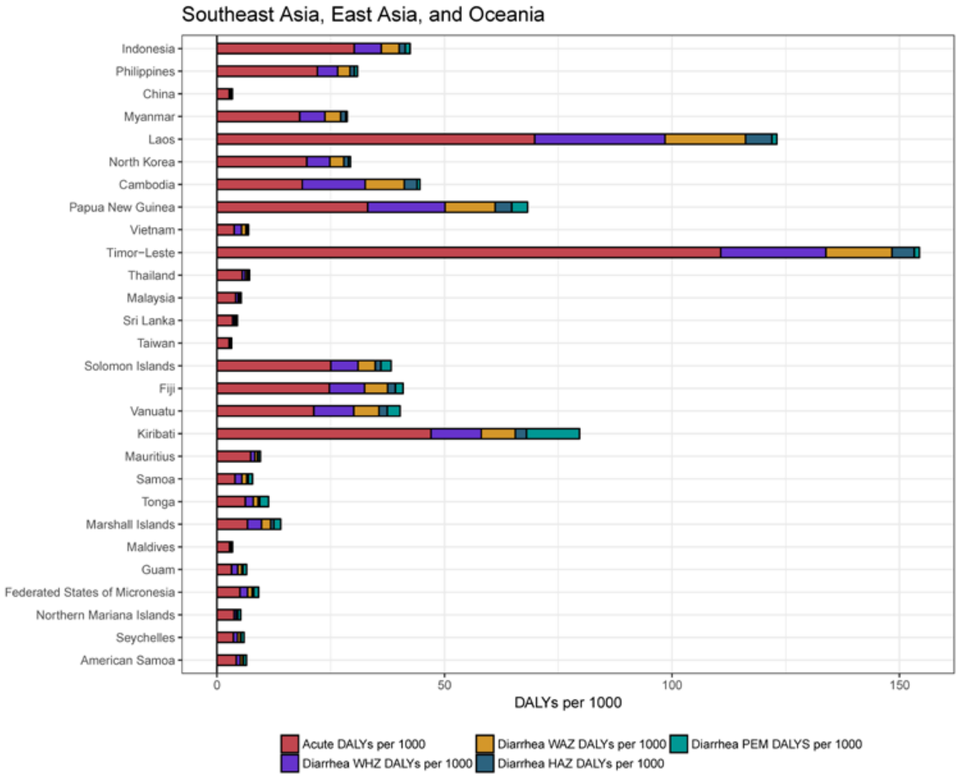
Southeast Asia, East Asia, and Oceania
Indonesia
Philippines
China
Myanmar
Laos
North Korea
Cambodia
Papua New Guinea
Vietnam
Timor−Leste
Thailand
Malaysia
Sri Lanka
Taiwan
Solomon Islands
Fiji
Vanuatu
Kiribati
Mauritius
Samoa
Tonga
Marshall Islands
Maldives
Guam
Federated States of Micronesia
Northern Mariana Islands
Seychelles
American Samoa
0
50
100
150
DALYs per 1000
Acute DALYs per 1000
Diarrhea WHZ DALYs per 1000
Diarrhea WAZ DALYs per 1000
Diarrhea HAZ DALYs per 1000
Diarrhea PEM DALYS per 1000

E

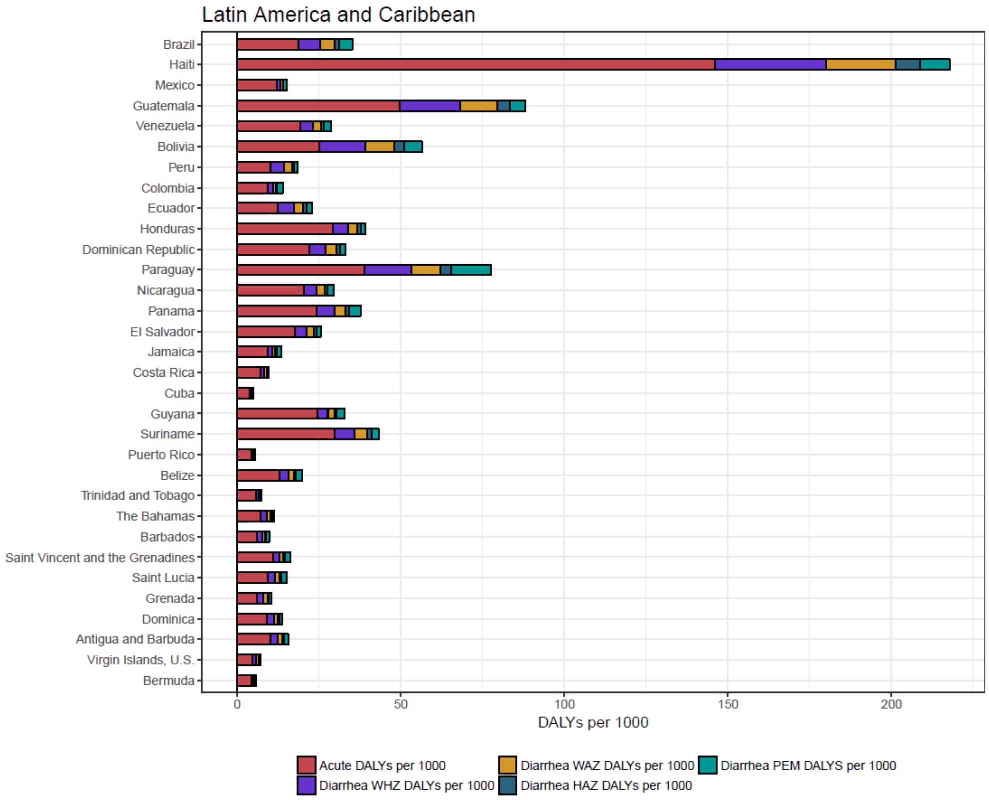
Latin America and Caribbean
Brazil
Haiti
Mexico
Guatemala
Venezuela
Bolivia
Peru
Colombia
Ecuador
Honduras
Dominican Republic
Paraguay
Nicaragua
Panama
El Salvador
Jamaica
Costa Rica
Cuba
Guyana
Suriname
Puerto Rico
Belize
Trinidad and Tobago
The Bahamas
Barbados
Saint Vincent and the Grenadines
Saint Lucia
Grenada
Dominica
Antigua and Barbuda
Virgin Islands, U.S.
Bermuda
0
50
100
150
200
DALYs per 1000
Acute DALYs per 1000
Diarrhea WHZ DALYs per 1000
Diarrhea WAZ DALYs per 1000
Diarrhea HAZ DALYs per 1000
Diarrhea PEM DALYS per 1000

455 **F**

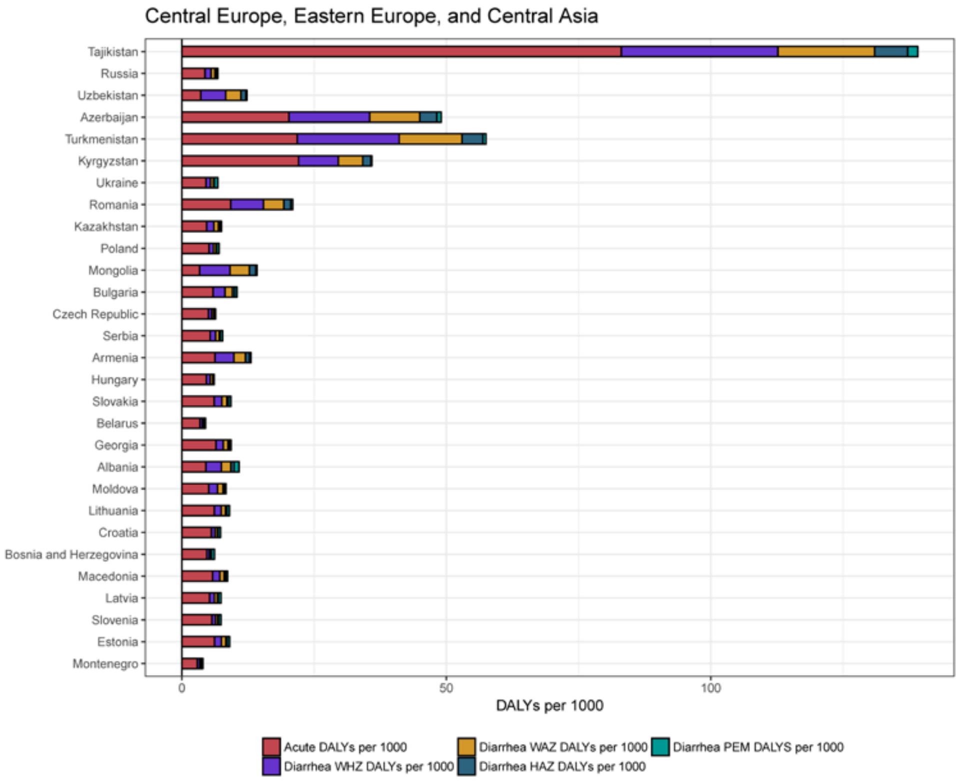

Central Europe, Eastern Europe, and Central Asia
Tajikistan
Russia
Uzbekistan
Azerbaijan
Turkmenistan
Kyrgyzstan
Ukraine
Romania
Kazakhstan
Poland
Mongolia
Bulgaria
Czech Republic
Serbia
Armenia
Hungary
Slovakia
Belarus
Georgia
Albania
Moldova
Lithuania
Croatia
Bosnia and Herzegovina
Macedonia
Latvia
Slovenia
Estonia
Montenegro
0
50
100
DALYs per 1000
Acute DALYs per 1000
Diarrhea WHZ DALYs per 1000
Diarrhea WAZ DALYs per 1000
Diarrhea HAZ DALYs per 1000
Diarrhea PEM DALYS per 1000

G

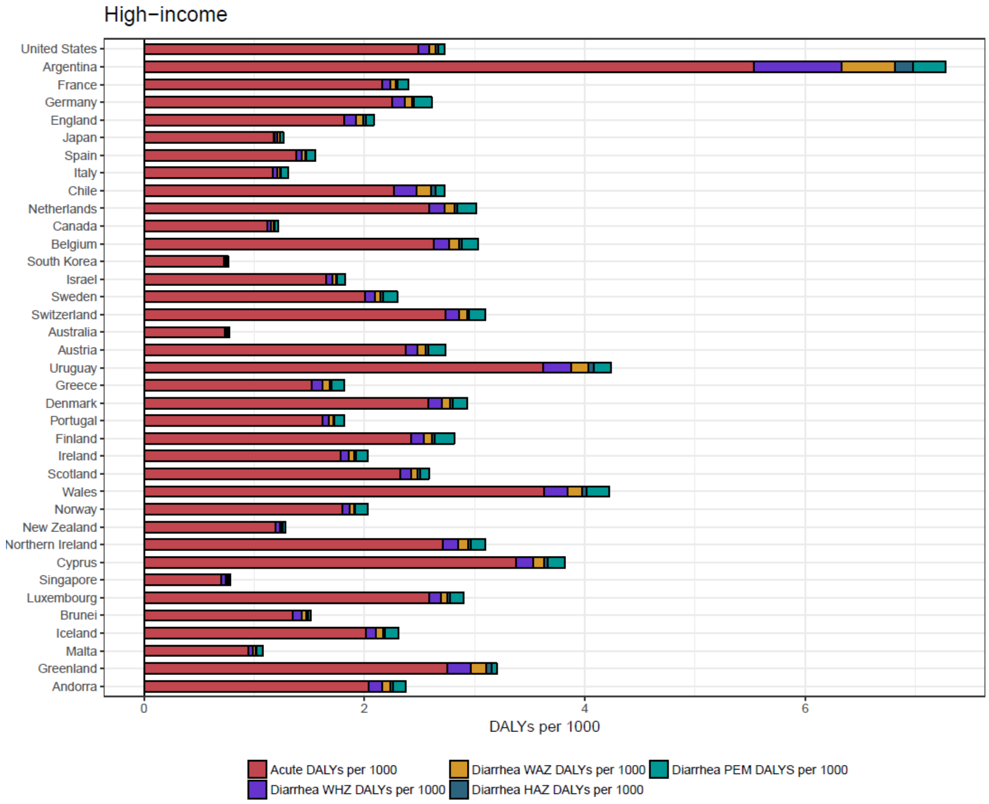
High−income
United States
Argentina
France
Germany
England
Japan
Spain
Italy
Chile
Netherlands
Canada
Belgium
South Korea
Israel
Sweden
Switzerland
Australia
Austria
Uruguay
Greece
Denmark
Portugal
Finland
Ireland
Scotland
Wales
Norway
New Zealand
Northern Ireland
Cyprus
Singapore
Luxembourg
Brunei
Iceland
Malta
Greenland
Andorra
0
2
4
6
DALYs per 1000
Acute DALYs per 1000
Diarrhea WAZ DALYs per 1000
Diarrhea PEM DALYS per 1000
Diarrhea WHZ DALYs per 1000
Diarrhea HAZ DALYs per 1000

References

1. GBD Diarrhoeal Diseases Collaborators. Estimates of global, regional, and national morbidity, mortality, and aetiologies of diarrhoeal diseases: a systematic analysis for the Global Burden of Disease Study 2015. *Lancet Infect Dis*. June 2017. doi:10.1016/S1473-3099(17)30276-1.

2. GBD 2016 Causes of Death Collaborators. Global, regional, and national age-sex specific mortality for 264 causes of death, 1980-2016: a systematic analysis for the Global Burden of Disease Study 2016. *Lancet Lond Engl*. 2017;390(10100):1151-1210. doi:10.1016/S0140-6736(17)32152-9.

3. GBD 2016 Disease and Injury Incidence and Prevalence Collaborators. Global, regional, and national incidence, prevalence, and years lived with disability for 328 diseases and injuries for 195 countries, 1990-2016: a systematic analysis for the Global Burden of Disease Study 2016. *Lancet Lond Engl*. 2017;390(10100):1211-1259. doi:10.1016/S0140-6736(17)32154-2.

4. GBD 2016 Risk Factors Collaborators. Global, regional, and national comparative risk assessment of 84 behavioural, environmental and occupational, and metabolic risks or clusters of risks, 1990-2016: a systematic analysis for the Global Burden of Disease Study 2016. *Lancet Lond Engl*. 2017;390(10100):1345-1422. doi:10.1016/S0140-6736(17)32366-8.

5. Foreman KJ, Lozano R, Lopez AD, Murray CJ. Modeling causes of death: an integrated approach using CODEm. *Popul Health Metr*. 2012;10:1. doi:10.1186/1478-7954-10-1.

6. GBD 2016 Disease and Injury Incidence and Prevalence Collaborators. Global, regional, and national incidence, prevalence, and years lived with disability for 328 diseases and injuries for 195 countries, 1990-2016: a systematic analysis for the Global Burden of Disease Study 2016. *Lancet Lond Engl*. 2017;390(10100):1211-1259. doi:10.1016/S0140-6736(17)32154-2.

7. Olofin I, McDonald CM, Ezzati M, Flaxman S, Black RE, Fawzi WW, Caulfield LE, Danaei G, Nutrition Impact Model Study (anthropometry cohort pooling). Associations of suboptimal growth with all-cause and cause-specific mortality in children under five years: a pooled analysis of ten prospective studies. *PloS One*. 2013;8(5):e64636. doi:10.1371/journal.pone.0064636.

8. GBD 2015 Risk Factors Collaborators. Global, regional, and national comparative risk assessment of 79 behavioural, environmental and occupational, and metabolic risks or clusters of risks, 1990-2015: a systematic analysis for the Global Burden of Disease Study 2015. *Lancet Lond Engl*. 2016;388(10053):1659-1724. doi:10.1016/S0140-6736(16)31679-8.

9. GBD 2015 LRI Collaborators. Estimates of the global, regional, and national morbidity, mortality, and aetiologies of lower respiratory tract infections in 195 countries: a systematic analysis for the Global Burden of Disease Study 2015. *Lancet Infect Dis*. August 2017. doi:10.1016/S1473-3099(17)30396-1.

10. World Health Organization. *WHO Child Growth Standards: Methods and Development*.; 2006. http://www.who.int/childgrowth/standards/technical_report/en/. Accessed July 27, 2016.

11. Kuczmarski R, Ogden C, Grummer-Strawn L, et al. *CDC Growth Charts: United States. Advance Data from Vital and Health Statistics*. Hyattsville, Maryland: National Center for Health Statistics http://www.cdc.gov/nchs/data/ad/ad314.pdf. Accessed August 23, 2016.

12. GBD 2015 Mortality and Causes of Death Collaborators. Global, regional, and national life expectancy, all-cause mortality, and cause-specific mortality for 249 causes of death, 1980-2015: a systematic analysis for the Global Burden of Disease Study 2015. *Lancet Lond Engl*. 2016;388(10053):1459-1544. doi:10.1016/S0140-6736(16)31012-1.

13. GBD 2015 Disease and Injury Incidence and Prevalence Collaborators. Global, regional, and national incidence, prevalence, and years lived with disability for 310 diseases and injuries, 1990-2015: a systematic analysis for the Global Burden of Disease Study 2015. *Lancet Lond Engl*. 2016;388(10053):1545-1602. doi:10.1016/S0140-6736(16)31678-6.

14. GBD 2015 Risk Factors Collaborators. Global, regional, and national comparative risk assessment of 79 behavioural, environmental and occupational, and metabolic risks or clusters of risks, 1990-2015: a systematic analysis for the Global Burden of Disease Study 2015. *Lancet Lond Engl*. 2016;388(10053):1659-1724. doi:10.1016/S0140-6736(16)31679-8.

15. Sudfeld CR, Charles McCoy D, Danaei G, Fink G, Ezzati M, Andrews KG, Fawzi WW. Linear Growth and Child Development in Low- and Middle-Income Countries: A Meta-Analysis. *Pediatrics*. 2015;135(5):e1266-e1275. doi:10.1542/peds.2014-3111.

16. Donowitz JR, Alam M, Kabir M, Ma JZ, Nazib F, Platts-Mills JA, Bartelt LA, Haque R, Petri WA. A Prospective Longitudinal Cohort to Investigate the Effects of Early Life Giardiasis on Growth and All Cause Diarrhea. *Clin Infect Dis*. June 2016:ciw391. doi:10.1093/cid/ciw391.

17. Faruque, ASG (PI). *Cryptosporidia Burden Study [Unpublished].* Dhaka, Bangladesh; 2016.

18. Kattula D, Sarkar R, Sivarathinaswamy P, Velusamy V, Venugopal S, Naumova EN, Muliyil J, Ward H, Kang G. The first 1000 days of life: prenatal and postnatal risk factors for morbidity and growth in a birth cohort in southern India. *BMJ Open*. 2014;4(7):e005404. doi:10.1136/bmjopen-2014-005404.

19. Kirkpatrick BD, Colgate ER, Mychaleckyj JC, Haque R, Dickson DM, Carmolli MP, Nayak U, Taniuchi M, Naylor C, Qadri F, Ma JZ, Alam M, Walsh MC, Diehl SA, Teams the PS, Petri WA. The “Performance of Rotavirus and Oral Polio Vaccines in Developing Countries” (PROVIDE) Study: Description of Methods of an Interventional Study Designed to Explore Complex Biologic Problems. *Am J Trop Med Hyg*. 2015;92(4):744-751. doi:10.4269/ajtmh.14-0518.

20. MAL-ED Network Investigators. The MAL-ED study: a multinational and multidisciplinary approach to understand the relationship between enteric pathogens, malnutrition, gut physiology, physical growth, cognitive development, and immune responses in infants and children up to 2 years of age in resource-poor environments. *Clin Infect Dis Off Publ Infect Dis Soc Am*. 2014;59 Suppl 4:S193-206. doi:10.1093/cid/ciu653.

21. Assis AMO, Barreto ML, Santos LMP, Fiaccone R, da Silva Gomes GS. Growth faltering in childhood related to diarrhea: a longitudinal community based study. *Eur J Clin Nutr*. 2005;59(11):1317–1323.

22. Bairagi R, CHOWDHURY MK, Kim YJ, T CURLIN G, GRAY RH. The association between malnutrition and diarrhoea in rural Bangladesh. *Int J Epidemiol*. 1987;16(3):477–481.

23. Becker S, Black RE, Brown KH. Relative effects of diarrhea, fever, and dietary energy intake on weight gain in rural Bangladeshi children. *Am J Clin Nutr*. 1991;53(6):1499-1503.

24. Black RE, Brown KH, Becker S. Effects of Diarrhea Associated with Specific Enteropathogens on the Growth of Children in Rural Bangladesh. *Pediatrics*. 1984;73(6):799-805.

25. Kotloff KL, Nataro JP, Blackwelder WC, Nasrin D, Farag TH, Panchalingam S, Wu Y, Sow SO, Sur D, Breiman RF, Faruque AS, Zaidi AK, Saha D, Alonso PL, Tamboura B, Sanogo D, Onwuchekwa U, Manna B, Ramamurthy T, Kanungo S, Ochieng JB, Omore R, Oundo JO, Hossain A, Das SK, Ahmed S, Qureshi S, Quadri F, Adegbola RA, Antonio M, Hossain MJ, Akinsola A, Mandomando I, Nhampossa T, Acácio S, Biswas K, O'Reilly CE, Mintz ED, Berkeley LY, Muhsen K, Sommerfelt H, Robins-Browne RM, Levine MM. Burden and aetiology of diarrhoeal disease in infants and young children in developing countries (the Global Enteric Multicenter Study, GEMS): a prospective, case-control study. *Lancet Lond Engl*. 2013;382(9888):209-222. doi:10.1016/S0140-6736(13)60844-2.

26. Lee G, Paredes Olortegui M, Peñataro Yori P, Black RE, Caulfield L, Banda Chavez C, Hall E, Pan WK, Meza R, Kosek M. Effects of Shigella-, Campylobacter- and ETEC-associated Diarrhea on Childhood Growth: *Pediatr Infect Dis J*. 2014;33(10):1004-1009. doi:10.1097/INF.0000000000000351.

27. Lima AAM, Moore SR, Barboza MS, Soares AM, Schleupner MA, Newman RD, Sears CL, Nataro JP, Fedorko DP, Wuhib T, others. Persistent diarrhea signals a critical period of increased diarrhea burdens and nutritional shortfalls: a prospective cohort study among children in northeastern Brazil. *J Infect Dis*. 2000;181(5):1643–1651.

28. MAL-ED Network Investigators. The MAL-ED study: a multinational and multidisciplinary approach to understand the relationship between enteric pathogens, malnutrition, gut physiology, physical growth, cognitive development, and immune responses in infants and children up to 2 years of age in resource-poor environments. *Clin Infect Dis Off Publ Infect Dis Soc Am*. 2014;59 Suppl 4:S193-206. doi:10.1093/cid/ciu653.

29. Moffat T. Diarrhea, respiratory infections, protozoan gastrointestinal parasites, and child growth in Kathmandu, Nepal. *Am J Phys Anthropol*. 2003;122(1):85-97. doi:10.1002/ajpa.10258.

30. Mølbak K, Andersen M, Aaby P, Højlyng N, Jakobsen M, Sodemann M, da Silva AP. Cryptosporidium infection in infancy as a cause of malnutrition: a community study from Guinea-Bissau, west Africa. *Am J Clin Nutr*. 1997;65(1):149-152.

31. Mondal D, Petri WA, Sack RB, Kirkpatrick BD, Haque R. Entamoeba histolytica-associated diarrheal illness is negatively associated with the growth of preschool children: evidence from a prospective study. *Trans R Soc Trop Med Hyg*. 2006;100(11):1032-1038. doi:10.1016/j.trstmh.2005.12.012.

32. Moore SR, Lima A a. M, Conaway MR, Schorling JB, Soares AM, Guerrant RL. Early childhood diarrhoea and helminthiases associate with long-term linear growth faltering. *Int J Epidemiol*. 2001;30(6):1457-1464. doi:10.1093/ije/30.6.1457.

33. Moore SR, Lima NL, Soares AM, Oriá RB, Pinkerton RC, Barrett LJ, Guerrant RL, Lima AAM. Prolonged Episodes of Acute Diarrhea Reduce Growth and Increase Risk of Persistent Diarrhea in Children. *Gastroenterology*. 2010;139(4):1156-1164. doi:10.1053/j.gastro.2010.05.076.

34. Moy RJ, de C Marshall TF, Choto RG, McNeish AS, Booth IW. Diarrhoea and growth faltering in rural Zimbabwe. *Eur J Clin Nutr*. 1994;48(11):810-821.

35. Prosper Sawadogo S, Martin-Prével Y, Capon G, Traoré SA, Delpeuch F. Nutritional status and common infections in infants in Burkina Faso: interest of an 'overall morbidity score': Infant nutritional status and common infections. *Trop Med Int Health*. 2008;13(3):345-353. doi:10.1111/j.1365-3156.2008.02008.x.

36. Walker SP, Grantham-McGregor SM, Powell CA, Himes JH, Simeon DT. Morbidity and the growth of stunted and nonstunted children, and the effect of supplementation. *Am J Clin Nutr*. 1992;56(3):504-510.

37. Weisz A, Meuli G, Thakwalakwa C, Trehan I, Maleta K, Manary M. The duration of diarrhea and fever is associated with growth faltering in rural Malawian children aged 6-18 months. *Nutr J*. 2011;10(1):25. doi:10.1186/1475-2891-10-25.

38. Wierzba TF, El-Yazeed RA, Savarino SJ, Mourad AS, Rao M, Baddour M, El-Deen AN, Naficy AB, Clemens JD. The interrelationship of malnutrition and diarrhea in a periurban area outside Alexandria, Egypt. *J Pediatr Gastroenterol Nutr*. 2001;32(2):189–196.

39. Altman DG, Bland JM. How to obtain the confidence interval from a P value. *BMJ*. 2011;343:d2090. doi:10.1136/bmj.d2090.

40. Olofin I, McDonald CM, Ezzati M, Flaxman S, Black RE, Fawzi WW, Caulfield LE, Danaei G, for the Nutrition Impact Model Study (anthropometry cohort pooling). Associations of Suboptimal Growth with All-Cause and Cause-Specific Mortality in Children under Five Years: A Pooled Analysis of Ten Prospective Studies. Wiley AS, ed. *PLoS ONE*. 2013;8(5):e64636. doi:10.1371/journal.pone.0064636.

41. Benichou J. A review of adjusted estimators of attributable risk. *Stat Methods Med Res*. 2001;10(3):195-216.

42. GBD 2015 DALYs and HALE Collaborators. Global, regional, and national disability-adjusted life-years (DALYs) for 315 diseases and injuries and healthy life expectancy (HALE), 1990–2015: a systematic analysis for the Global Burden of Disease Study 2015 - PIIS0140-6736(16)31460-X.pdf. *The Lancet*. 2016;388:1603-1658.

43. Lamberti LM, Fischer Walker CL, Black RE. Systematic review of diarrhea duration and severity in children and adults in low- and middle-income countries. *BMC Public Health*. 2012;12:276. doi:10.1186/1471-2458-12-276.
